# Polygenic risk for triglyceride levels in the presence of a high impact rare variant

**DOI:** 10.1186/s12920-023-01717-2

**Published:** 2023-11-08

**Authors:** Shengjie Ying, Tracy Heung, Bhooma Thiruvahindrapuram, Worrawat Engchuan, Yue Yin, Christina Blagojevic, Zhaolei Zhang, Robert A. Hegele, Ryan K. C. Yuen, Anne S. Bassett

**Affiliations:** 1 Institute of Medical Science, University of Toronto, Toronto, ON Canada; 2 Schulich School of Medicine and Dentistry, Western University, London, ON Canada; 3 Clinical Genetics Research Program, Centre for Addiction and Mental Health, Toronto, ON Canada; 4 The Dalglish Family 22Q Clinic, University Health Network, Toronto, ON Canada; 5 The Centre for Applied Genomics, The Hospital for Sick Children, Toronto, ON Canada; 6 Department of Molecular Genetics, University of Toronto, Toronto, ON Canada; 7 Donnelly Centre for Cellular and Biomolecular Research, University of Toronto, Toronto, ON Canada; 8 Department of Computer Science, University of Toronto, Toronto, ON Canada; 9 Department of Psychiatry, University of Toronto, Toronto, ON Canada; 10 Toronto General Hospital Research Institute and Campbell Family Mental Health Research Institute, Toronto, ON Canada

**Keywords:** Triglyceride, Lipid, Polygenic risk score, 22q11.2 microdeletion, Genome sequencing

## Abstract

**Background:**

Elevated triglyceride (TG) levels are a heritable and modifiable risk factor for cardiovascular disease and have well-established associations with common genetic variation captured in a polygenic risk score (PRS). In young adulthood, the 22q11.2 microdeletion conveys a 2-fold increased risk for mild-moderate hypertriglyceridemia. This study aimed to assess the role of the TG-PRS in individuals with this elevated baseline risk for mild-moderate hypertriglyceridemia.

**Methods:**

We studied a deeply phenotyped cohort of adults (*n* = 157, median age 34 years) with a 22q11.2 microdeletion and available genome sequencing, lipid level, and other clinical data. The association between a previously developed TG-PRS and TG levels was assessed using a multivariable regression model adjusting for effects of sex, BMI, and other covariates. We also constructed receiver operating characteristic (ROC) curves using logistic regression models to assess the ability of TG-PRS and significant clinical variables to predict mild-moderate hypertriglyceridemia status.

**Results:**

The TG-PRS was a significant predictor of TG-levels (*p* = 1.52E-04), along with male sex and BMI, in a multivariable model (p_model_ = 7.26E-05). The effect of TG-PRS appeared to be slightly stronger in individuals with obesity (BMI ≥ 30) (beta = 0.4617) than without (beta = 0.1778), in a model unadjusted for other covariates (*p*-interaction = 0.045). Among ROC curves constructed, the inclusion of TG-PRS, sex, and BMI as predictor variables produced the greatest area under the curve (0.749) for classifying those with mild-moderate hypertriglyceridemia, achieving an optimal sensitivity and specificity of 0.746 and 0.707, respectively.

**Conclusions:**

These results demonstrate that in addition to significant effects of sex and BMI, genome-wide common variation captured in a PRS also contributes to the variable expression of the 22q11.2 microdeletion with respect to elevated TG levels.

**Supplementary Information:**

The online version contains supplementary material available at 10.1186/s12920-023-01717-2.

## Background

Elevated circulating triglyceride (TG) levels are a well-established risk factor for cardiovascular disease (CVD), with accumulating evidence supporting a causal role [1–3]. Other CVD-associated lipid profiles include elevated low density lipoprotein cholesterol (LDLC) and total cholesterol (TC), and low levels of high density lipoprotein cholesterol (HDLC) [4, 5]. The genetic architecture of lipid levels is polygenic [6], comprising rare variants with high penetrance (e.g., *LDLR* mutations in familial hypercholesterolemia) [7, 8], and the aggregate effects of common variation that can be captured in a polygenic risk score (PRS) [9, 10].

Among the genetic contributors to lipid disorders are rare copy number variants (CNVs). Classically, these have been confined to CNVs that impact a single well-established dyslipidemia gene (e.g., *LDLR*, *LPL*) [11]. Recently, however, we demonstrated that the 22q11.2 microdeletion confers an approximately 2-fold increased risk for mild-moderate hypertriglyceridemia (HTG; TG levels 1.7–10.0 mmol/L) compared to general population risk [12]. Related conditions also associated with the 22q11.2 deletion include type 2 diabetes [13] (T2D) and obesity [14]. To our knowledge, this is the only recurrent multigenic CNV to be associated with elevated TG levels that does not overlap an established TG metabolism gene. The 22q11.2 microdeletion, with estimated live birth prevalence of 1 in 2148 [15], defines the 22q11.2 deletion syndrome (22q11.2DS), and has proven utility as a genetic model to study associated common complex conditions [16]. Its role as a genetic model includes serving as a platform to investigate the interplay between rare and common genetic variation, an area that has become of intense research interest and potential clinical relevance [17–22]. For example, recent studies have demonstrated that additional genome-wide variation, rare CNVs and schizophrenia PRS, can contribute to likelihood of schizophrenia expression, where there is baseline > 20-fold increased risk conferred by the 22q11.2 deletion [18, 22, 23].

In this study, we aimed to assess the additional genomic and phenotypic contributors to lipid levels (TG, HDLC, LDLC, TC) in individuals with a 22q11.2 microdeletion, with a focus on TG levels given the associated elevated baseline risk for mild-moderate HTG [12]. We studied a unique, deeply-phenotyped adult cohort of individuals with 22q11.2DS where there were both lipid level and genome sequencing data available for this rare condition (Additional file 1: Figure S1). The main goals of the study were to test whether lipid PRSs derived from the general population are associated with lipid levels (TG, LDLC, HDLC, and TC) in individuals with a 22q11.2 microdeletion. In exploratory post-hoc analyses, we constructed receiver operating characteristic (ROC) curves to assess the predictive value of the TG-PRS and other clinical variables for mild-moderate HTG status. We also examined the role of additional genome-wide clinically relevant rare variants, and assessed the contribution of overall candidate gene-based rare variants, on lipid levels.

## Methods

### 22q11.2DS cohort and clinical variables

This study involved a well-characterized cohort of adults with a typical 22q11.2 microdeletion ascertained from a specialized 22q11.2DS clinic in Toronto, Canada. Typical 22q11.2 deletions were identified through standard clinical laboratory methods [13, 24] and precise 22q11.2 deletion extents confirmed using genome sequencing data (see Additional file 1: Table S1 for details).

To be included, participants had to have at least one recorded circulating lipid level of TC, TG, LDLC, and/or HDLC (Additional File 1: Figure S1), obtained from routine clinical bloodwork assessments. Measurements were taken predominantly in the non-fasting but not post-prandial state, as this was most feasible for this patient population [12]. For most individuals we used their most recent bloodwork. LDLC levels were calculated using the Friedewald equation. However, in cases where LDLC levels were unavailable due to high TG levels that result in an inaccurate estimation by Friedewald equation [25], we used records of LDLC levels at other time points when available. No LDLC levels were calculated using the Friedewald equation when TG levels were > 4.52 mmol/L, consistent with previous lipid genetics studies [9, 26].

Additionally, we assessed other traits known to influence lipid levels or genetic background, including sex, age, BMI, T2D, psychotic illness [27, 28], and ancestry. T2D was defined as having a hemoglobin A1c value ≥ 6.5% and/or diagnosed with T2D as indicated by medical records. We defined “psychotic” as individuals diagnosed with schizophrenia or schizoaffective disorder; all other individuals were deemed “non-psychotic”. European versus non-European ancestry was assigned using principal component analysis (PCA) of common genetic variants (Additional file 1: Figure S2), which showed complete concordance with pedigree-derived information.

For details on genome sequencing methods and variant annotation, see Additional file 2: Supplementary Methods.

### Polygenic risk score analyses

We used lipid PRSs that were previously constructed in a UK Biobank study [29] (PGS Catalog publication ID: PGP000263), with a development sample of 391,124 European individuals using the penalized regression (bigstatsr) method. Genotype positions and effect sizes for the TG (PGS001979), HDLC (PGS001954), LDLC (PGS001933), and TC (PGS001895) PRSs were retrieved from the PGS catalog [30] (Additional file 1: Table S3). Individual-level PRSs for the study cohort were calculated using PRSice-2 following QC (Additional File 1: Figure S3).

We tested for associations between PRSs and their corresponding lipid level using linear regression in 1) a univariable model and 2) a multivariable model that adjusted for other key phenotypic variables, batch (TCAG vs IBBC cohort and sequencing platform), and the first four principal components (PC) of ancestry.

- 1) lipid level ~ lipid PRS

- 2) lipid level ~ lipid PRS + sex* + age + BMI + T2D* + psychotic illness* + cohort + sequencing platform + PC1–PC4 *binary variable

Binary variables were coded as 0 or 1 and all values were standardized using the scale() function in R to produce standardized beta coefficients. For regression analyses, TG levels were natural log transformed to approximate a normal distribution, as done previously [8, 9, 31] (Additional File 1: Figure S4). For individuals on statins, LDLC and TC levels were divided by 0.7 and 0.8, respectively, to adjust for the cholesterol-lowering effects of these medications, as done previously [8, 9, 32]. The variance in lipid level explained by each multivariable model was measured using the multiple R^2^ metric. The variance in lipid levels explained by the PRS variable alone in a multivariable model (i.e., ΔR^2^) was calculated as the difference in the multiple R^2^ between the multivariable model when including the PRS variable (full model) versus without the PRS variable (covariate only model). Additionally, we tested for an interaction between TG-PRS x BMI by adding this interaction variable to a model that included TG-PRS and BMI as other independent variables and to the multivariable model (2) (Additional file 1: Table S4).

### Receiver operating characteristic (ROC) curve analyses

Given the elevated baseline risk for mild-moderate HTG for individuals with 22q11.2DS, we constructed ROC curves to classify mild-moderate HTG status based on logistic regression models using TG-PRS, sex, and BMI as predictor variables, independently or in various combinations (TG-PRS + BMI, TG-PRS + sex, BMI + sex, TG-PRS + BMI + sex). Logistic regression models were implemented using the glm() function in R and all visualizations and analyses related ROC curves were done using the R package “pROC” [33]. Delong’s test for two correlated ROC curves was used to test for the difference between the area under the curve (AUC) of two ROC curves and the optimal sensitivity and specificity of each ROC curve was determined using Youden’s J statistic. Confidence intervals for AUCs were calculated using 2000 bootstrap replicates.

### Rare variant analyses

To prioritize variants for assessment of clinical relevance with respect to their relationship to causing extreme lipid levels (i.e., high TG, LDLC, HDLC, and low HDLC), we restricted to variants affecting protein coding or splicing regions that are (1) very rare (gnomAD PopMax filtering allele frequency < 0.2%), (2) loss of function (LoF) or predicted damaging missense, and (3) within genes relevant to lipid levels that are part of a targeted next generation sequencing (NGS) panel (*n* = 33 candidate genes) used at a specialized genetics clinic for lipid metabolism disorders in London, Ontario [34] (Additional file 1: Table S5). Prioritized rare variants were then assessed using the American College of Medical Genetics and Genomics (ACMG) variant interpretation guidelines [35] or *LDLR*-specific guidelines developed by ClinGen [36]. For further details on variant prioritization, see Additional File 2: Supplementary methods.

Additionally, we sought to assess whether being a carrier of a rare variant, including those with potentially smaller effect sizes that are not considered pathogenic/likely pathogenic per ACMG criteria, would be associated with altered lipid levels (Additional file 1: Table S5). An association between “rare variant carrier status” and lipid levels was assessed using the same univariable and multivariable linear regression models as for PRS analyses, but with the rare variant carrier status variable in place of the PRS variable. For additional details on the filtering criteria for rare variants for this analysis, see Additional File 2: Supplementary Methods.

All statistical analyses were performed using R version 4.0.3. Statistical significance was defined as *p* < 0.05. *P*-values were not adjusted for multiple testing.

## Results

### Cohort description and demographic and phenotypic predictors of lipid levels

Table 1 summarizes the clinical and demographic features of the 151 of 157 individuals with a 22q11.2 microdeletion who had genome sequencing data that passed common variant quality control (Additional file 1: Figure S1). As expected from previous results for an overlapping sample (Additional file 1: Table S2), 45.0% of the cohort with TG data available (*n*=67 of 149) had mild-moderate HTG (TG 1.7–10.0 mmol/L), representing an approximately twofold increase in risk compared to an age-matched general Canadian population prevalence of 21.6% [12]. Males in particular had a significantly higher prevalence of HTG (60.8% vs 29.3%, Fisher’s exact test *p* = 1.44E-04) and significantly lower average HDLC levels (0.99 mmol/L vs 1.31 mmol/L, Wilcoxon test *p* = 3.02E-09) (Table 1).

**Table 1:** Lipid and other phenotypic/clinical variables, and sex effects, in adults with a 22q11.2 microdeletion

***Continuous variables***									
	**Total sample**			**Males**			**Females**		
	**n**	**Mean**	**SD**	**n**	**Mean**	**SD**	**n**	**Mean**	**SD**
**Lipid levels**									
TG (mmol/L)^a^	149	1.92	1.18	74	2.25	1.37	75	1.60	0.85
HDLC (mmol/L)	150	1.15	0.35	75	0.99	0.28	75	1.31	0.33
LDLC (mmol/L)	148	2.71	0.87	73	2.67	0.86	75	2.75	0.88
LDLC statin-adjusted (mmol/L)^b^	148	2.81	0.93	73	2.74	0.85	75	2.88	0.99
TC (mmol/L)	151	4.72	0.98	76	4.64	0.98	75	4.80	0.99
TC statin-adjusted (mmol/L)^b^	151	4.84	1.06	76	4.74	1.02	75	4.94	1.09
**Other clinical/demographic variables**									
BMI (kg/m^2^)	151	30.29	7.39	76	29.82	7.36	75	30.77	7.44
Age^c^	151	35.66	11.2	76	34.92	11.51	75	36.42	10.9
***Categorical variables***									
	**Total sample (*n* = 151)**			**Males (*n* = 76)**			**Females (*n* = 75)**		
	**n**		**%**	**n**		**%**	**n**		**%**
Mild-moderate HTG^a,d^	67		45.0	45		60.8	22		29.3
**Other clinical/demographic variables**									
On statin medication	16		10.6	7		9.2	9		12.0
Type 2 diabetes	14		9.3	8		10.5	6		8.0
Psychotic illness^e^	65		43.0	37		48.7	28		37.3
European ancestry	135		89.4	71		93.4	64		85.3

*SD* Standard deviation, *TG* Triglyceride, *LDLC* Low density lipoprotein cholesterol, *HDLC* High density lipoprotein cholesterol, *TC* Total cholesterol, *BMI* Body mass index, *HTG* Hypertriglyceridemia, *T2D* type 2 diabetes

^a^Excluded one individual on fibrates (*n* = 1)

^b^For individuals on statin medications, LDLC and TC levels were divided by 0.7 and 0.8, respectively, as in previous studies [8, 9, 32]

^c^Recorded at the date of TC measurement to the nearest 0.1 years

^d^Mild-moderate HTG is defined as having a TG level between 1.7–10 mmol/L. For this row only, there are *n* = 74 males and *n* = 149 total

^e^Defined as schizophrenia or schizoaffective disorder

### Polygenic risk score and other predictors of lipid levels

We first examined whether lipid PRSs (for TG, HDLC, LDLC, and TC) that were previously developed in an entirely European general population cohort (aged 40–69 years) [29] (Additional file 1: Table S3), would be significantly associated with their corresponding lipid level in a relatively younger (median age 34, range 17–64, years), predominantly (89.4%) European, and smaller (*n* = 149–151) cohort of individuals with a 22q11.2 microdeletion. The results showed that each PRS was a significant predictor (all *p* < 0.01) of its corresponding lipid level, in univariable models. Multivariable linear regression models that adjusted for sex, age, BMI, T2D, psychotic illness, ascertainment cohort, sequencing platform, and the first four principal components (PCs) of ancestry (Table 2, Additional file 1: Figure S7) were significant however only for TG and HDLC levels; overall models were non-significant for LDLC and TC (only the PRS variable appeared to have a significant effect). Results were similar when restricting to only individuals of European ancestry (*n* = 134–136), with no notable changes in effect sizes of the respective PRSs (Additional file 1: Table S6).

**Table 2:** Linear regression analyses testing lipid polygenic risk score (PRS) as a predictor of its corresponding lipid level, in a univariable model and in a multivariable model accounting for phenotypic, batch, and ancestry variables

	**TG (*n* = 149)**^a^			**HDLC (*n* = 150)**			**LDLC (*n* = 148)**^b^			**TC (*n* = 151)**^b^		
	**beta**	**std error**	**p**	**beta**	**std error**	**p**	**beta**	**std error**	**p**	**beta**	**std error**	**p**
**Univariable model**												
PRS (for the lipid level assessed)	0.2403	0.0803	**0.0032**	0.4579	0.0737	**5.00E-09**	0.2403	0.0805	**0.0034**	0.2262	0.0809	**0.0058**
**Multivariable model**												
PRS (for the lipid level assessed)	0.3133	0.0804	**1.52E-04**	0.3818	0.0667	**6.28E-08**	0.2524	0.0839	**0.0031**	0.2494	0.0851	**0.0040**
Female sex	-0.2633	0.0771	**8.36E-04**	0.3980	0.0669	**2.17E-08**	0.0717	0.0862	0.4067	0.0818	0.0853	0.3392
Age	0.0019	0.0849	0.9820	0.0602	0.0721	0.4050	0.0777	0.0940	0.4099	0.1237	0.0939	0.1899
BMI (kg/m^2^)	0.2944	0.0803	**3.51E-04**	-0.3009	0.0673	**1.61E-05**	0.1013	0.0881	0.2522	0.1040	0.0881	0.2398
T2D	-0.0642	0.0819	0.4343	0.0590	0.0697	0.3990	-0.0328	0.0928	0.7247	-0.0001	0.0908	0.9989
Psychotic illness^c^	0.0472	0.0792	0.5527	-0.0025	0.0669	0.9710	0.0347	0.0885	0.6957	0.0068	0.0870	0.9380
Originating ascertainment cohort	-0.0676	0.1559	0.6654	0.1313	0.1329	0.3250	0.0608	0.1727	0.7255	-0.0471	0.1728	0.7857
Sequencing platform	0.1972	0.1230	0.1112	-0.1072	0.1047	0.3080	-0.0629	0.1388	0.6512	0.0402	0.1369	0.7697
Ancestry PC1	-0.0827	0.0787	0.2953	0.0078	0.0663	0.9060	-0.0659	0.0870	0.4503	-0.0476	0.0870	0.5852
Ancestry PC2	-0.1035	0.0989	0.2970	0.1073	0.0823	0.1950	0.0811	0.1073	0.4509	0.0823	0.1072	0.4439
Ancestry PC3	0.1311	0.1407	0.3532	-0.0049	0.1195	0.9680	-0.0847	0.1555	0.5868	-0.1042	0.1557	0.5043
Ancestry PC4	0.0366	0.0853	0.6687	-0.0755	0.0730	0.3030	-0.0808	0.0942	0.3926	-0.1176	0.0944	0.2151
	**R**^**2**^		**p**	**R**^**2**^		**p**	**R**^**2**^		**p**	**R**^**2**^		**p**
**Model**	0.2471		**7.26E-05**	0.4565		**2.46E-13**	0.0914		0.3400	0.0909		0.3258
	**ΔR**^**2**^			**ΔR**^**2**^			**ΔR**^**2**^			**ΔR**^**2**^		
**PRS variable**	0.0843			0.1302			0.0647			0.0630		

Beta coefficients are standardized with positive values indicating a positive association between higher lipid levels and female sex, older age, higher BMI, having type 2 diabetes, having a psychotic illness, belonging to the TCAG sequencing project cohort, sequenced using a HiSeq X, and higher value for PC (and thus a negative beta value indicates a negative association for that variable, i.e., lower lipid level)

ΔR^2^, here an estimate of the variance in each lipid level explained by the PRS, is calculated by subtracting the R^2^ of the multivariable model without the PRS variable (i.e., using all remaining phenotypic, batch, and ancestry variables) from the R^2^ of the full model

*TG* Triglyceride, *LDLC* Low density lipoprotein cholesterol, *HDLC* High density lipoprotein cholesterol, *TC* Total cholesterol, *BMI* Body mass index, *T2D* Type 2 diabetes, *PC* Principal component for ancestry

Bold font indicates statistical significance

^a^Excluded one individual on fibrate treatment. Triglyceride levels were natural log transformed to approximate a normal distribution

^b^For individuals on statin medications, LDLC and TC levels were divided by 0.7 and 0.8, respectively, as in previous studies [8, 9, 32]

^c^Defined as schizophrenia or schizoaffective disorder

The TG-PRS variable alone (beta = 0.313, *p* = 1.52E-04) explained 8.4% of the variance (ΔR^2^) in TG levels in the multivariable model (*R*^2^_model_ = 24.7%, p_model_ = 7.26E-05) (Table 2), with male sex and higher BMI also significant independent predictors of higher TG levels (Table 2).

**Fig. 1:**
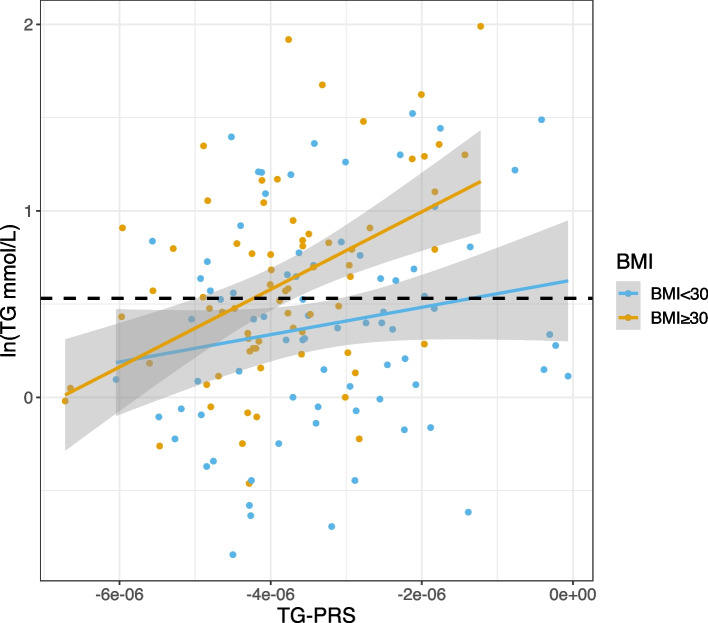
Scatterplot and linear associations between the triglyceride (TG) polygenic risk score (TG-PRS) and TG levels for *n* = 149 adults with a 22q11.2 microdeletion by obesity classification. Fitted lines were generated using linear regression performed within two sub-groups, with (orange) (beta = 0.4617) and without (blue) (beta = 0.1778) obesity defined as BMI ≥ 30 kg/m^2^ (unadjusted TG x BMI interaction beta = 0.179, *p* = 0.045). TG levels are natural log (2ln) transformed. The dashed black line indicates the natural log transformed value of the lower-bound clinical cut-off that defines mild-moderate HTG (1.7 mmol/L), thus above this line individuals would be classified as having mild-moderate hypertriglyceridemia

Univariable linear regression analyses within those with or without obesity (BMI ≥ 30), revealed that the effect size of the association between the TG-PRS and TG levels was greater in those with obesity (beta = 0.4617) than without obesity (beta = 0.1778) (Fig. 1). Further testing for a difference between these effect sizes (i.e., the slope of the regression lines), by adding an interaction term (TG-PRS x BMI) to a linear regression model that included TG-PRS and BMI as predictors of TG levels, identified a significant interaction (beta = 0.179, *p* = 0.045) (Additional file 1: Table S4). The interaction term did not reach significance however when adjusted for the other 10 variables included in the multivariable models (beta = 0.168, *p* = 0.059) (Additional file 1: Table S4).

### Using triglyceride polygenic risk score (TG-PRS), BMI, and sex to classify mild-moderate hypertriglyceridemia

**Fig. 2:**
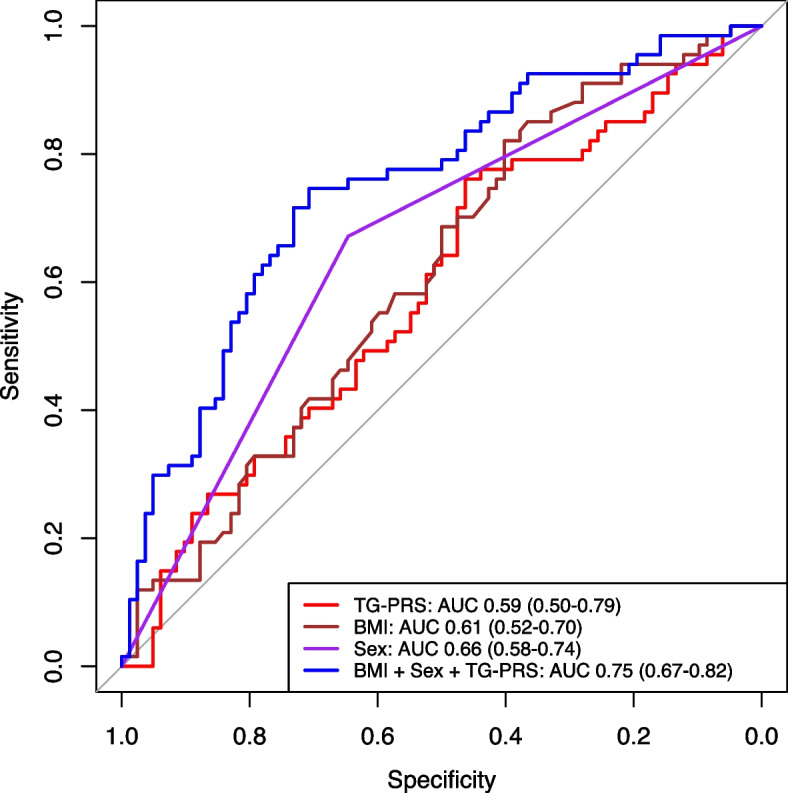
Receiver operating characteristic (ROC) curves of logistic regression models using each of triglyceride polygenic risk score (TG-PRS; red), BMI (brown), sex (purple), and the combination of these three variables (blue), as predictors of mild-moderate hypertriglyceridemia in adults with 22q11.2DS. The area under the curve (AUC) and 95% confidence intervals (95% CI) for each curve are shown in the figure key on the bottom right

In post-hoc analyses we constructed ROC curves using logistic regression models to assess the ability of the TG-PRS, along with significant clinical predictors of TG levels (sex and BMI), to discriminate between those with and without clinically defined mild-moderate HTG (TG levels 1.7–10.0 mmol/L). Among the univariable models, sex had the highest AUC (0.659; 95% CI 0.582–0.736), with BMI and TG-PRS having slightly lower AUCs (Fig. 2, Additional File 1: Table S7). Combining all three variables in one model resulted in a significantly higher AUC (0.7486) than each univariable model (vs TG-PRS *p* = 0.0023, vs BMI *p* = 0.0089, vs sex *p* = 0.0024), achieving an optimal sensitivity and specificity (using the Youden Index) of 0.746 and 0.707, respectively (Additional File 1: Tables S7 and S8). Furthermore, we tested whether the addition of TG-PRS to each clinical predictor alone (i.e., sex vs sex + TG-PRS, BMI vs BMI + TG-PRS) or combined (i.e., sex + BMI vs sex + BMI + TG-PRS) would improve prediction (Additional File 1: Figure S8). In each case, the addition of TG-PRS marginally increased the AUC, but the difference did not reach statistical significance (Additional File 1: Table S8).

We also assessed the prevalence of mild-moderate HTG within each decile of TG-PRS (Additional File 1: Figure S9). Individuals in the top decile had a non-significantly higher prevalence of mild-moderate HTG (64.3%; *n* = 9 of 14) compared to individuals in the lowest decile (26.7%, *n* = 4 of 15) (Fisher’s exact test *p* = 0.3166).

### Clinically relevant rare variants

In 32 (20.5%) of 156 individuals we identified 38 rare (< 0.2%) single nucleotide (SNV) or insertion/deletion (indels) variants in 17 of the 33 lipid panel genes assessed, which we then examined using standard clinical criteria [35, 36] (Additional file 1: Table S9). There were no rare CNVs overlapping exonic regions of these 33 genes (Additional file 1: Table S5).

Of these 38 SNV/indels, two were classified as pathogenic/likely pathogenic, one for hypercholesterolemia and the other for HTG. A pathogenic heterozygous missense variant in *LDLR* (c.G523A, p.D175N; rs121908033; ClinVar ID: 3726) [37, 38], diagnostic for familial hypercholesterolemia, was identified in a 21.1-year-old male with an elevated LDLC level of 4.53 mmol/L. We also identified a loss of function (LoF) (frameshift insertion) variant in *CREB3L3* (c.729dupG, p.L243fs; rs780374391; ClinVar ID: 967,101) that was classified as likely pathogenic with respect to mild-moderate HTG. This female patient had a variable history of elevated TG levels with a maximum recorded TG level of 3.89 mmol/L (age 46.9 years), and most recent recorded TG level of 1.57 mmol/L (age 47.4 years). See Additional file 1: Table S10 for detailed patient history and scoring of these two variants.

### Rare variant carrier status regression analyses

Of the *n* = 154–156 individuals assessed for the four lipid traits examined (Additional file 1: Figure S1), we identified relatively few individuals who were carriers of rare (< 1.0%) variants in genes canonically associated with each lipid trait: high TG (*n* = 7, two individuals had two variants each), high LDLC (*n* = 14), low HDLC (*n* = 9), and high HDLC (*n* = 10) (Additional file 1: Table S5, Table S11). Rare variant carrier status was not a significant predictor of the level of corresponding lipid trait using either univariable or multivariable linear regression models (Additional file 1: Table S12), possibly due to limited power afforded by the sample size.

## Discussion

In this initial study of lipid genetics in individuals with the most common pathogenic CNV in humans, we demonstrate that part of the variable expression of the 22q11.2 microdeletion with respect to elevated TG levels can be explained by genome-wide common variation captured in a PRS, along with significant effects of sex and BMI. With few individuals having rare variants in TG metabolism genes, this study was underpowered to determine their role in 22q11.2DS.

The modifying effect on TG levels from the TG-PRS in the presence of a high impact “first-hit” exerted by the 22q11.2 deletion is consistent with previous studies of schizophrenia and related neuropsychiatric phenotypes in 22q11.2DS, where Schizophrenia-PRS has been reported to modify the associated penetrance of the deletion [18, 39]. This pattern for the 22q11.2 deletion also appears to be consistent with previously reported PRS modification of the BMI-lowering effect of the 16p11.2 duplication [40]. In other conditions, PRSs have also been reported to modify the penetrance of a pathogenic rare variant affecting a single gene, e.g., in familial hypercholesterolemia [21, 40], coronary artery disease [17], breast cancer [20], and prostate cancer [20]. Notably, the variance in TG levels explained by the TG-PRS (8%) in a multivariable model in the current study of 22q11.2DS appears in line with general population expectations for TG-PRS explaining variance in TG levels (~ 2–10%), although it is difficult to make head-to-head comparisons of PRS performance given that each study used a different PRS and different methods to quantify PRS performance [41–43].

Consistent with previous studies using general population samples [44–47], in this 22q11.2 microdeletion sample we identified a potential interaction between TG-PRS and BMI, in an unadjusted interaction model, that indicates that the TG-PRS may have a stronger association with TG levels amongst individuals with obesity. This suggests that obesity may play a role in further unmasking the TG level-increasing effect of a high TG-PRS, potentially further increasing the likelihood of HTG in individuals with a 22q11.2 microdeletion who also have elevated BMI.

The potential value of PRSs as a clinical risk prediction tool is a widely debated topic [48, 49]. In a previous study of PRS and schizophrenia in 22q11.2DS, it was suggested that PRSs may potentially have greater clinical use when there is an elevated baseline risk, as stratification using PRSs would be able to produce larger differences in absolute risk [39]. In this study, we assessed the ability of the TG-PRS, along with significant clinical variables, sex and BMI, to predict mild-moderate HTG, in the context of an elevated baseline risk conveyed by a typical pathogenic 22q11.2 deletion. While the performance of each of these three variables independently was relatively poor (AUC 0.59–0.61), the combination of the three demonstrated moderate predictive value (AUC = 0.75, sensitivity = 0.75, specificity = 0.71). However, the addition of the TG-PRS to each clinical variable-only model did not demonstrate a significant increase prediction accuracy, suggesting that the TG-PRS used adds no predictive value that would be clinically meaningful to that obtained from standard clinical data. Furthermore, while we observed a substantial difference in the prevalence of mild-moderate HTG between individuals in the highest (64.3%) vs the lowest (26.7%) deciles of TG-PRS, this difference did not reach statistical significance, likely related to the relatively small size of each decile bin (*n* = 14–15) and the effect size of the PRS used.

It is also important to consider when an intermediate biomarker, such as a lipid PRS, would be clinically useful. Lipid PRSs are predictors of lipid levels, which are risk factors for end-point diseases, such as coronary artery disease [6]. Lipid PRSs would not serve as actionable markers in adults where lipid levels are obtainable from routine bloodwork, as the lipid level itself would guide management. Future research may provide a potential use-case for 22q11.2DS in childhood, prior to HTG manifestation, if a well-validated and more highly predictive TG-PRS was shown that could further motivate preventive measures, such as physical exercise and healthy diet, for those individuals with high TG-PRS.

The results of this study demonstrate the potential value of a sample of individuals with a rare clinically relevant CNV where there is both deep phenotyping and genome sequencing data available to study complex, polygenic disorders, especially those of high clinical relevance. To our knowledge, this study contains the largest available cohort of adults with a 22q11.2 microdeletion and lipid level and other essential phenotypic data that enabled an assessment of the added value of lipid PRSs and combined ability with phenotypic data for predicting HTG. This study would not be possible using current large-scale biobank data, as there is an acknowledged selection bias against individuals with rare high-impact CNVs (e.g., *n* = 10 with a pathogenic 22q11.2 deletion in UK BioBank) [50, 51]. Sequencing data enabled the identification of two rare pathogenic/likely pathogenic variants relevant to lipid disorders.

This study also has several limitations. Due to the rarity of 22q11.2DS, especially with relevant adult data, the sample size is relatively small. This limited the ability to compare mild-moderate HTG prevalence between extremes of the PRS distribution (e.g., highest vs lowest decile of TG-PRS), and to further assess effects of rare variant burden on lipid levels [8, 10]. Also, given the sample size limitations, we opted to include individuals of non-European ancestry in the main analyses and accounted for ancestry using PCA, despite applying a PRS developed using only individuals of European ancestry where the predictive performance may be decreased by up to half [29] when applied to non-European ancestries. Although we observed no substantial differences in effect sizes when restricting to only individuals of European ancestry, using a PRS derived from a multi-ancestry GWAS [9] may yield better overall performance. Also, we were unable to assess the influence of lifestyle/environmental factors on TG levels; this will require future studies.

## Conclusions

In conclusion, we found that the TG-PRS is associated with TG levels in the context of elevated baseline risk for mild-moderate HTG conferred by the 22q11.2 microdeletion. The results contribute to the body of literature demonstrating how additional genome-wide common variation can modify the expression of a high-impact rare variant.

### Supplementary Information

**Additional file 1: Table S1.** Deletion extent details of individuals with a typical pathogenic 22q11.2 deletion included in the main polygenic risk score analyses (n_max_=151). **Table S2.** Comparison of lipid and other clinical and demographic variables between the adult 22q11.2DS cohort included in the main PRS analyses in this study (maximum *n*=151) and in Blagojevic et al. (2022) [1] (*n*=267) (*n*=125 overlapping). **Table S3.** Polygenic risk scores developed in Privé et al. 2022 [3] (PGS ID: PGP000263) for each lipid trait, retrieved from the PGS catalog [4]. **Table S4.** Linear regression models testing an interaction between TG-PRS and BMI in adults with a 22q11.2 microdeletion. **Table S5.** Genes associated with each lipid level that are included in the Dron et al. 2020 [2] NGS panel. This table is adapted from Dron et al. 2020 [2]. **Table S6.** Within individuals of European ancestry with a 22q11.2 microdeletion, linear regression analyses testing lipid polygenic risk score (PRS) as a predictor of its corresponding lipid level in a univariable model and in a multivariable model accounting for phenotypic, batch, and ancestry variables. **Table S7.** Measures related to receiver operating characteristic (ROC) curves based on logistic regression models for mild-moderate hypertriglyceridemia (triglyceride level 1.7–10.0 mmol/L; outcome variable) in adults with 22q11.2DS using variables in the “Predictor variables” column. **Table S8.** Comparison of the area under the curve (AUC) between two receiver operating characteristic (ROC) curves constructed using logistic regression models that predict mild-moderate hypertriglyceridemia status (triglyceride level 1.7–10.0 mmol/L) (outcome variable) in adults with 22q11.2DS. **Table S9.** Thirty-eight very rare (PopMax FAF<0.2%) LoF or predicted deleterious missense heterozygous variants in 32 individuals with 22q11.2DS prioritized for assessment of clinical relevance with respect to extreme levels of TG, LDLC or HDLC. **Table S10.** Variant assessments based on ACMG/AMP guidelines for the two variants classified as pathogenic/likely pathogenic. **Table S11.** Variants (frequency<1.0%) in adults with 22q11.2DS included in the rare variant burden analyses. **Table S12.** Linear regression analyses testing lipid trait-specific rare carrier status as a predictor for its corresponding lipid level in a univariable model and in a multivariable model accounting for phenotypic, batch, ancestry variables, in adults with 22q11.2DS. **Figure S1. **Overview of study design. **Figure S2.** First two principal components of individuals with 22q11.2DS from the TCAG (*n*=88) and IBBC (*n*=63) cohorts, and individuals from 1000 Genomes with assigned ancestry. A cut-off of -0.02 on the PC2 axis was used to assign individuals in this study (TCAG or IBBC) as European (<-0.02) or non-European (>0.02). **Figure S3.** Distribution of polygenic risk scores (PRS) for triglycerides (TG) (*n*=149), high density lipoprotein cholesterol (HDLC) (*n*=150), low density lipoprotein cholesterol (LDLC) (*n*=148), and total cholesterol (TC) (*n*=151). **Figure S4**. Distribution of triglyceride levels in individuals with a 22q11.2 deletion (*n*=149), before and after log transformation. **Figure S5.** Number of rare variants called per individual between the three site-platform groups, by variant type, for *n*=151 individuals included in the rare variant burden analyses. The mean ± SD number of variants across all three groups (*n*=151) is displayed next to each plot title. Variant count means between the three site-platform groups were compared using the Kruskal–Wallis test and pairwise comparisons were made using Wilcoxon signed-rank without adjustment for multiple comparisons. Samples were sequenced to average ± standard deviation (SD) read depths of 29.36 ± 8.42 (IBBC-HiSeq 2500), 37.22 ± 10.90 (IBBC-HiSeq X) and 94.58 ± 23.33 (TCAG-HiSeq X). The read depths reported for the TCAG-HiSeq X samples are the combined read depths of the proband and both parents. **Figure S6.** Lipid levels of individuals from each site-platform group for *n*=151 individuals included in the polygenic risk score and rare variant burden analyses. Lipid levels between the three site-platform groups per trait were compared using the Kruskal-Wallis test and pairwise comparisons were made using Wilcoxon signed-rank without adjustment for multiple comparisons. TG, triglyceride; LDLC, low density lipoprotein cholesterol; HDLC, high density lipoprotein cholesterol; TC, total cholesterol. **Figure S7.** Linear association between the polygenic risk score (common variants) for each lipid trait and its corresponding lipid level. Fitted lines were generated using linear regression performed within sex. TG, triglyceride; LDLC, low density lipoprotein cholesterol; HDLC, high density lipoprotein cholesterol; TC, total cholesterol; PRS, polygenic risk score. **Figure S8.** Receiver operating characteristic (ROC) curves of logistic regression models predicting mild-moderate hypertriglyceridemia in adults with 22q11.2DS. Each plot contains two ROC curves. One contains only one or two clinical variable(s) as predictors: BMI (A), sex (B) or both (C). The other curve includes the TG-PRS with the respective clinical variable(s). The area under the curve (AUC) and 95% confidence intervals (95% CI) for each curve are shown in the figure key on the bottom right of each plot. **Figure S9.** The proportion of adult individuals with 22q11.2DS and (A) mild-moderate hypertriglyceridemia (HTG) and (B) the triglyceride (TG) level (mmol/L) for individuals in each decile of triglyceride polygenic risk score (TG-PRS) (*n*=14–15 per decile; *n*=149 total). The dashed line in B indicates the lower-bound cut-off for mild-moderate HTG (1.7 mmol/L).

**Additional file 2. **Supplementary Methods.

## Data Availability

The datasets generated and/or analysed during the current study are not publicly available due to the sensitive nature of the detailed clinical and genotype data but are available from the corresponding author on reasonable request.
