# Correction: Polygenic risk for triglyceride levels in the presence of a high impact rare variant

**DOI:** 10.1186/s12920-023-01742-1

**Published:** 2023-11-23

**Authors:** Shengjie Ying, Tracy Heung, Bhooma Thiruvahindrapuram, Worrawat Engchuan, Yue Yin, Christina Blagojevic, Zhaolei Zhang, Robert A. Hegele, Ryan K. C. Yuen, Anne S. Bassett

**Affiliations:** 1 Institute of Medical Science, University of Toronto, Toronto, ON Canada; 2 Schulich School of Medicine and Dentistry, Western University, London, ON Canada; 3 Clinical Genetics Research Program, Centre for Addiction and Mental Health, Toronto, ON Canada; 4 The Dalglish Family 22Q Clinic, University Health Network, Toronto, ON Canada; 5 The Centre for Applied Genomics, The Hospital for Sick Children, Toronto, ON Canada; 6 Department of Molecular Genetics, University of Toronto, Toronto, ON Canada; 7 Donnelly Centre for Cellular and Biomolecular Research, University of Toronto, Toronto, ON Canada; 8 Department of Computer Science, University of Toronto, Toronto, ON Canada; 9 Department of Psychiatry, University of Toronto, Toronto, ON Canada; 10 Toronto General Hospital Research Institute and Campbell Family Mental Health Research Institute, Toronto, ON Canada

**Correction: BMC Med Genomics 16, 281 (2023)**

**https://doi.org/10.1186/s12920-023-01717-2**

Following publication of the original article [1], the authors reported errors in Table 1. The first heading of the table should read “Continuous variables” and not “Categorical variables”. The correct table is given in this correction article. The original article [1] has been corrected.

**Table 1:** Lipid and other phenotypic/clinical variables, and sex effects, in adults with a 22q11.2 microdeletion

***Continuous*** ***variables***									
	**Total sample**			**Males**			**Females**		
	**n**	**Mean**	**SD**	**n**	**Mean**	**SD**	**n**	**Mean**	**SD**
**Lipid levels**									
TG (mmol/L)^a^	149	1.92	1.18	74	2.25	1.37	75	1.60	0.85
HDLC (mmol/L)	150	1.15	0.35	75	0.99	0.28	75	1.31	0.33
LDLC (mmol/L)	148	2.71	0.87	73	2.67	0.86	75	2.75	0.88
LDLC statin-adjusted (mmol/L)^b^	148	2.81	0.93	73	2.74	0.85	75	2.88	0.99
TC (mmol/L)	151	4.72	0.98	76	4.64	0.98	75	4.80	0.99
TC statin-adjusted (mmol/L)^b^	151	4.84	1.06	76	4.74	1.02	75	4.94	1.09
**Other clinical/demographic variables**									
BMI (kg/m^2^)	151	30.29	7.39	76	29.82	7.36	75	30.77	7.44
Age^c^	151	35.66	11.2	76	34.92	11.51	75	36.42	10.9
***Categorical*** ***variables***									
	**Total sample (*n* = 151)**			**Males (*n* = 76)**			**Females (*n* = 75)**		
	**n**		**%**	**n**		**%**	**n**		**%**
Mild-moderate HTG^a,d^	67		45.0	45		60.8	22		29.3
**Other clinical/demographic variables**									
On statin medication	16		10.6	7		9.2	9		12.0
Type 2 diabetes	14		9.3	8		10.5	6		8.0
Psychotic illness^e^	65		43.0	37		48.7	28		37.3
European ancestry	135		89.4	71		93.4	64		85.3

*SD* Standard deviation, *TG* Triglyceride, *LDLC* Low density lipoprotein cholesterol, *HDLC* High density lipoprotein cholesterol, *TC* Total cholesterol, *BMI* Body mass index, *HTG* Hypertriglyceridemia, *T2D* type 2 diabetes

^a^Excluded one individual on fibrates (*n* = 1)

^b^For individuals on statin medications, LDLC and TC levels were divided by 0.7 and 0.8, respectively, as in previous studies [8, 9, 32]

^c^Recorded at the date of TC measurement to the nearest 0.1 years

^d^Mild-moderate HTG is defined as having a TG level between 1.7–10 mmol/L. For this row only, there are *n* = 74 males and *n* = 149 total

^e^Defined as schizophrenia or schizoaffective disorder
