# Supplementary material for: Polygenic risk for triglyceride levels in the presence of a high impact rare variant
Source: BMC Med Genomics. 2023 Nov 8;16:281. doi: 10.1186/s12920-023-01717-2 (PMC10634078; doi:10.1186/s12920-023-01717-2)
Supplement: Supplementary file 1 — Additional file 1: Table S1. Deletion extent details of individuals with a typical pathogenic 22q11.2 deletion included in the main polygenic risk score analyses (nmax=151). Table S2. Comparison of lipid and other clinical and demographic variables between the adult 22q11.2DS cohort included in the main PRS analyses in this study (maximum n=151) and in Blagojevic et al. (2022) [1] (n=267) (n=125 overlapping). Table S3. Polygenic risk scores developed in Privé et al. 2022 [3] (PGS ID: PGP000263) for each lipid trait, retrieved from the PGS catalog [4]. Table S4. Linear regression models testing an interaction between TG-PRS and BMI in adults with a 22q11.2 microdeletion. Table S5. Genes associated with each lipid level that are included in the Dron et al. 2020 [2] NGS panel. This table is adapted from Dron et al. 2020 [2]. Table S6. Within individuals of European ancestry with a 22q11.2 microdeletion, linear regression analyses testing lipid polygenic risk score (PRS) as a predictor of its corresponding lipid level in a univariable model and in a multivariable model accounting for phenotypic, batch, and ancestry variables. Table S7. Measures related to receiver operating characteristic (ROC) curves based on logistic regression models for mild-moderate hypertriglyceridemia (triglyceride level 1.7–10.0 mmol/L; outcome variable) in adults with 22q11.2DS using variables in the “Predictor variables” column. Table S8. Comparison of the area under the curve (AUC) between two receiver operating characteristic (ROC) curves constructed using logistic regression models that predict mild-moderate hypertriglyceridemia status (triglyceride level 1.7–10.0 mmol/L) (outcome variable) in adults with 22q11.2DS. Table S9. Thirty-eight very rare (PopMax FAF<0.2%) LoF or predicted deleterious missense heterozygous variants in 32 individuals with 22q11.2DS prioritized for assessment of clinical relevance with respect to extreme levels of TG, LDLC or HDLC. Table S10. Variant assessments bas [file 12920_2023_1717_MOESM1_ESM.docx]

**ADDITIONAL FILE 1: SUPPLEMENTARY DATA**

**Table S1.** Deletion extent details of individuals with a typical pathogenic 22q11.2 deletion included in the main polygenic risk score analyses (n_max_=151).

| **Typical 22q11.2 deletion extents** | **n (total n=151)** | **%** |
| --- | --- | --- |
| LCRA-LCRD | 143 | 94.7 |
| LCRA-LCRB | 7 | 4.6 |
| LCRA-LCRC | 1 | 0.7 |

**Table S2**. Comparison of lipid and other clinical and demographic variables between the adult 22q11.2DS cohort included in the main PRS analyses in this study (maximum n=151) and in Blagojevic et al. (2022)[1] (n=267) (n=125 overlapping).

|  | **Blagojevic et al. 2022** | | | **This study** | | | **Wilcoxon test** | | | |
| --- | --- | --- | --- | --- | --- | --- | --- | --- | --- | --- |
|  | **n** | **Mean** | **SD** | **n** | **Mean** | **SD** | **p** | | | |
| **Continuous variables** |  |  |  |  |  |  |  | | | |
| TG (mmol/L) | 267 | 1.75 | 1.14 | 149 | 1.92 | 1.18 | 0.0760 | | | |
| HDLC (mmol/L) | 267 | 1.18 | 0.35 | 150 | 1.15 | 0.35 | 0.4124 | | | |
| LDLC (mmol/L) | 260 | 2.62 | 0.79 | 148 | 2.71 | 0.87 | 0.3416 | | | |
| TC (mmol/L) | 267 | 4.58 | 0.93 | 151 | 4.72 | 0.98 | 0.1707 | | | |
| BMI | 267 | 29.29 | 7.79 | 151 | 30.29 | 7.39 | 0.0844 | | | |
| Age | 267 | 30.90 | 10.57 | 151 | 35.66 | 11.20 | 1.05E-05 | | | |
| Age exclusive^a^ | 142 | 27.33 | 9.42 | 26 | 42.02 | 13.30 | 2.31E-07 | | | |
| **Categorical variables** |  | | |  | | |  | | | |
|  | **Blagojevic et al. 2022 (n=267)** | | | **This study (n=151)** | | | **Fisher’s exact test** | | | |
|  | **n** | | **%** | **n** | | **%** | **OR** | **95% CI** | | **p** |
| Mild-moderate HTG^b^ | 107 | | 40.1 | 67 | | 45.0 | 0.82 | 0.54 | 1.25 | 0.3519 |
| Male | 131 | | 86.8 | 76 | | 50.3 | 0.95 | 0.63 | 1.45 | 0.8389 |
| Antipsychotic medication^c^ | 100 | | 66.2 | 74 | | 49.0 | 0.67 | 0.44 | 1.02 | 0.0529 |

TG, triglyceride; LDLC, low density lipoprotein cholesterol; HDLC, high density lipoprotein cholesterol; TC, total cholesterol; BMI, body mass index; OR, odds ratio; 95% CI, 95% confidence interval; HTG, hypertriglyceridemia.

^a^Individuals exclusive (non-overlapping) to each study.

^b^Mild-moderate HTG is defined as having a TG level between 1.7–10.0 mmol/L. For this row only, there are n=149 total in the column under the heading “This study”.

^c^Antipsychotic medication use was used as a proxy for psychotic illness in Blagojevic et al. 2022 [1].

**Table S3.** Polygenic risk scores developed in Privé et al. 2022[3] (PGS ID: PGP000263) for each lipid trait, retrieved from the PGS catalog[4].

| **Lipid trait** | **PGS ID** | **Original number of variants** | **Number of variants applied in this study^a^** |
| --- | --- | --- | --- |
| TG | PGS001979 | 71,203 | 70,684 |
| HDLC | PGS001954 | 85,429 | 84,781 |
| LDLC | PGS001933 | 25,604 | 25,084 |
| TC | PGS001895 | 16,576 | 16,284 |

TG, triglyceride; LDLC, low density lipoprotein cholesterol; HDLC, high density lipoprotein cholesterol; TC, total cholesterol

^a^Number of variants included in the PRSs following common variant QC (see methods) and exclusion of variants in the 22q11.2 deletion region (chr22:18,912,231–21,465,672 [GRCh37]).

**Table S4.** Linear regression models testing an interaction between TG-PRS and BMI in adults with a 22q11.2 microdeletion.

|  | **TG (n=149)^a^** | | |
| --- | --- | --- | --- |
| **Unadjusted interaction model** |  |  |  |
|  | **beta** | **std error** | **p** |
| PRS | 0.3306 | 4.1410 | **5.84E-05** |
| BMI | 0.2860 | 3.7140 | **2.91E-04** |
| PRS x BMI | 0.1787 | 2.0230 | **0.0449** |
|  | **R^2^** | | **p** |
| Model | 0.1647 | | **8.73E-06** |
| **Adjusted interaction model** |  |  |  |
| PRS | 0.3545 | 4.3010 | **3.24E-05** |
| Sex | -0.2439 | -3.1760 | **0.0019** |
| Age | -0.0024 | -0.0300 | 0.9765 |
| BMI | 0.2782 | 3.5060 | **6.18E-04** |
| T2D | -0.0491 | -0.6290 | 0.5304 |
| Psychotic^b^ | 0.0424 | 0.5410 | 0.5894 |
| Cohort | -0.0895 | -0.5800 | 0.5626 |
| Sequencing Platform | 0.2306 | 1.8680 | 0.0640 |
| PC1 | -0.0835 | -1.0710 | 0.2862 |
| PC2 | -0.1253 | -1.2700 | 0.2063 |
| PC3 | 0.1347 | 0.9670 | 0.3355 |
| PC4 | 0.0372 | 0.4410 | 0.6603 |
| PRS x BMI | 0.1675 | 1.9060 | 0.0588 |
|  | **R^2^** | | **p** |
| Model | 0.2668 | | **3.64E-05** |

TG, triglyceride; BMI, body mass index; T2D, Type 2 diabetes; PC, principal component.

All values were standardized using the scale() function in R prior to calculating the interaction terms and performing the regression analysis.

^a^Excluded individual on fibrates (n=1). Triglyceride levels were natural log transformed.

^b^Defined as schizophrenia or schizoaffective disorder.

Bold font indicates statistical significance.

Table S5. Genes associated with each lipid level that are included in the Dron et al. 2020[2] NGS panel. This table is adapted from Dron et al. 2020[2]

| **Lipid trait** | **Gene** | **Related monogenic disorder** | **Monogenic inheritance pattern (autosomal)** |
| --- | --- | --- | --- |
| **High LDLC** | ***LDLR*** | FH | Co-Dominant |
|  | ***APOB*** | FH^a^ | Co-Dominant |
|  | ***PCSK9*** | FH^b^ | Co-Dominant |
|  | *APOE** | FH | Dominant |
|  | *STAP1* | FH | Dominant |
|  | *LDLRAP1* | FH | Recessive |
|  | *LIPA* | FH | Recessive |
|  | *ABCG5* | Sitosterolemia & FH | Recessive |
|  | *ABCG8* | Sitosterolemia & FH | Recessive |
|  | *SORT1*^c^ | - |  |
|  | *MYLIP*^c^ | - |  |
| **High TG** | ***LPL*** | Severe HTG | Recessive |
|  | ***APOC2*** | Severe HTG | Recessive |
|  | ***APOA5*** | Severe HTG | Recessive |
|  | ***LMF1*** | Severe HTG | Recessive |
|  | ***GPIHBP1*** | Severe HTG | Recessive |
|  | *GPD1* | Transient infantile HTG | Recessive |
|  | *APOE** | Dysbetalipoproteinemia | Recessive |
|  | *GALNT2*^c^ | - |  |
|  | *MLXIPL*^c^ | - |  |
|  | *APOA4*^d^ | - |  |
|  | *PPARA*^d^ | - |  |
|  | *GCKR*^ce^ | - |  |
|  | *CREB3L3*^e^ | - |  |
|  | *TRIB1* | - |  |
| **Low HDLC** | ***ABCA1*** | Tangier disease | Recessive |
|  | ***APOA1*** | Primary hypoalphalipoproteinemia | Recessive |
|  | ***LCAT*** | Lecithin:cholesterol acyltransferase deficiency | Recessive |
|  | *PLTP*^d^ | - |  |
|  | *ABCG1*^d^ | - |  |
| **High HDLC** | ***CETP*** | Hyperalphalipoproteinemia | Recessive |
|  | ***LIPG*** | Hyperalphalipoproteinemia | Recessive |
|  | ***LIPC*** | Hepatic lipase deficiency | Recessive |
|  | ***SCARB1*** | SR-B1 deficiency | Recessive |

Genes considered “canonically” associated with the lipid trait are bolded. TG, triglyceride; LDLC, low density lipoprotein cholesterol; HDLC, high density lipoprotein cholesterol.

^a^Loss of function variants in *APOB* may also cause decreased levels of LDLC.

^b^Only gain of function variants is *PCSK9* are associated with higher levels of LDLC, whereas loss of function variants are associated with higher LDLC levels.

^c^Gene identified as a candidate through genome-wide association study results.
^d^Gene identified as a candidate through functional studies.
^e^Gene identified as a candidate through rare variant association.
**APOE* is present more than once in the table.

**Table S6.** Within individuals of European ancestry with a 22q11.2 microdeletion, linear regression analyses testing lipid polygenic risk score (PRS) as a predictor of its corresponding lipid level in a univariable model and in a multivariable model accounting for phenotypic, batch, and ancestry variables.

|  | **TG (n=134)^a^** | | | **HDLC (n=135)** | | | **LDLC (n=133)^b^** | | | **TC (n=136)^b^** | | |
| --- | --- | --- | --- | --- | --- | --- | --- | --- | --- | --- | --- | --- |
|  | **beta** | **std error** | **p** | **beta** | **std error** | **p** | **beta** | **std error** | **p** | **beta** | **std error** | **p** |
| **Univariable model** | |  |  |  |  |  |  |  |  |  |  |  |
| PRS | 0.2408 | 0.0842 | **0.0049** | 0.4697 | 0.0794 | **2.63E-08** | 0.2474 | 0.0841 | **0.0039** | 0.2261 | 0.0831 | **0.0073** |
| **Multivariable model** | |  |  |  |  |  |  |  |  |  |  |  |
| PRS | 0.2731 | 0.0841 | **0.0015** | 0.3658 | 0.0727 | **1.68E-06** | 0.2371 | 0.0887 | **0.0085** | 0.2138 | 0.0864 | **0.0147** |
| Sex | -0.2373 | 0.0805 | **0.0038** | 0.4102 | 0.0709 | **5.75E-08** | 0.1054 | 0.0916 | 0.2518 | 0.1397 | 0.0869 | 0.1104 |
| Age | 0.0486 | 0.0916 | 0.5969 | 0.0613 | 0.0788 | 0.4380 | 0.1423 | 0.1036 | 0.1723 | 0.1898 | 0.0985 | 0.0562 |
| BMI | 0.2649 | 0.0843 | **0.0021** | -0.3229 | 0.0719 | **1.61E-05** | 0.0621 | 0.0942 | 0.5112 | 0.0352 | 0.0906 | 0.6984 |
| T2D | -0.0943 | 0.0885 | 0.2889 | 0.0647 | 0.0761 | 0.3970 | -0.0267 | 0.1028 | 0.7956 | 0.0005 | 0.0956 | 0.9962 |
| Psychotic^c^ | -0.0308 | 0.0856 | 0.7197 | 0.0009 | 0.0726 | 0.9900 | -0.0089 | 0.0968 | 0.9267 | -0.0758 | 0.0910 | 0.4061 |
| Cohort | -0.1207 | 0.3970 | 0.7617 | 0.3479 | 0.3488 | 0.3210 | -0.0087 | 0.4459 | 0.9845 | -0.1749 | 0.4275 | 0.6831 |
| Sequencing Platform | 0.1727 | 0.1272 | 0.1770 | -0.0975 | 0.1095 | 0.3750 | -0.0489 | 0.1459 | 0.7380 | 0.0391 | 0.1384 | 0.7781 |
| PC1 | -0.0376 | 0.9195 | 0.9675 | 1.1026 | 0.7856 | 0.1630 | 0.7805 | 1.0249 | 0.4478 | 0.8133 | 0.9605 | 0.3988 |
| PC2 | 0.0940 | 0.9784 | 0.9236 | 0.4497 | 0.8457 | 0.5960 | 0.2174 | 1.1015 | 0.8439 | 0.4759 | 1.0555 | 0.6529 |
| PC3 | 0.0899 | 0.3934 | 0.8196 | 0.3579 | 0.3421 | 0.2970 | -0.0055 | 0.4413 | 0.9901 | 0.0021 | 0.4241 | 0.9961 |
| PC4 | 0.1190 | 0.3160 | 0.7072 | 0.0864 | 0.2728 | 0.7520 | 0.1626 | 0.3599 | 0.6522 | 0.3008 | 0.3414 | 0.3800 |
|  | **R^2^** | | **p** | **R^2^** | | **p** | **R^2^** | | **p** | **R^2^** | | **p** |
| Model | 0.2125 |  | **0.0027** | 0.4626 |  | **7.34E-12** | 0.1028 |  | 0.3306 | 0.1259 |  | 0.1418 |
|  | **ΔR^2^** | | | **ΔR^2^** | | | **ΔR^2^** | | | **ΔR^2^** | | |
| PRS variable | 0.0708 | | | 0.1120 | | | 0.0583 | | | 0.0528 | | |

TG, triglyceride; LDLC, low density lipoprotein cholesterol; HDLC, high density lipoprotein cholesterol; TC, total cholesterol; BMI, body mass index; T2D, Type 2 diabetes; PC, principal component.

Beta coefficients are standardized with positive values indicating a positive association between higher lipid levels and female sex, older age, higher BMI, having type 2 diabetes, having a psychotic illness, belonging to the TCAG sequencing project cohort, sequenced using a HiSeq X, and higher value for PC.

ΔR^2^, here an estimate of the variance in each lipid level explained by the PRS, is calculated by subtracting the R^2^ of the multivariable model without the PRS variable (i.e., using all remaining phenotypic, batch, and ancestry variables) from the R^2^ of the full model.

^a^Excluded one individual on fibrate treatment. Triglyceride levels were natural log transformed to approximate a normal distribution.

^b^For individuals on statin medications, LDLC and TC levels were divided by 0.7 and 0.8, respectively.

^c^Defined as schizophrenia or schizoaffective disorder.

Bold font indicates statistical significance.

**Table S7.** Measures related to receiver operating characteristic (ROC) curves based on logistic regression models for mild-moderate hypertriglyceridemia (triglyceride level 1.7–10.0 mmol/L; outcome variable) in adults with 22q11.2DS using variables in the “Predictor variables” column.

| **Predictor variables** | **AUC** | **95% CI (DeLong's method)** | | **Optimal threshold (Youden's method)** | **Youden index** | **Sensitivity^a^** | **Specificity^a^** | **Accuracy^a^** |
| --- | --- | --- | --- | --- | --- | --- | --- | --- |
| TG-PRS | 0.5897 | 0.4977 | 0.6818 | 0.4183 | 1.2246 | 0.7612 | 0.4634 | 0.5973 |
| BMI | 0.6124 | 0.5222 | 0.7026 | 0.3965 | 1.2233 | 0.8209 | 0.4024 | 0.5906 |
| Sex | 0.6590 | 0.5821 | 0.7359 | 0.4507 | 1.3180 | 0.6716 | 0.6463 | 0.6577 |
| TG-PRS + Sex | 0.7011 | 0.6171 | 0.7852 | 0.5307 | 1.3369 | 0.6418 | 0.6951 | 0.6711 |
| TG-PRS + BMI | 0.6340 | 0.5443 | 0.7236 | 0.4567 | 1.2068 | 0.5970 | 0.6098 | 0.6040 |
| BMI + Sex | 0.7214 | 0.6399 | 0.8030 | 0.5052 | 1.3790 | 0.6716 | 0.7073 | 0.6913 |
| BMI + Sex + TG-PRS | 0.7486 | 0.6692 | 0.8281 | 0.4397 | 1.4536 | 0.7463 | 0.7073 | 0.7248 |

TG, triglyceride; BMI, body mass index; PRS, polygenic risk score; AUC, area under the curve.

^a^The optimal threshold for sensitivity, specificity, and accuracy was determined using Youden’s J statistic.

**Table S8.** Comparison of the area under the curve (AUC) between two receiver operating characteristic (ROC) curves constructed using logistic regression models that predict mild-moderate hypertriglyceridemia status (triglyceride level 1.7–10.0 mmol/L) (outcome variable) in adults with 22q11.2DS.

| **ROC 1^a^** | **ROC 2^a^** | **AUC 1** | **AUC 2** | **Z^b^** | **p^b^** |
| --- | --- | --- | --- | --- | --- |
| sex + BMI + TG-PRS | TG-PRS | 0.7486 | 0.5897 | 3.0531 | 0.0023 |
| sex + BMI + TG-PRS | BMI | 0.7486 | 0.6124 | 2.6146 | 0.0089 |
| sex + BMI + TG-PRS | sex | 0.7486 | 0.6590 | 3.0410 | 0.0024 |
| BMI + TG-PRS | BMI | 0.6340 | 0.6124 | 0.5619 | 0.5742 |
| sex + TG-PRS | sex | 0.7011 | 0.6590 | 1.9343 | 0.0531 |
| sex + BMI + TG-PRS | sex + BMI | 0.7486 | 0.7214 | 1.3827 | 0.1667 |

TG, triglyceride; BMI, body mass index; PRS, polygenic risk score; ROC, receiver operating characteristic; AUC, area under the curve.

^a^Under each ROC column are the predictor variables for each logistic regression model. AUC 1 and AUC 2 are the corresponding AUCs of ROC 1 and ROC 2, respectively.

**^b^**Delong’s test for two correlated ROC curves was used to test for the difference between the AUC of two ROC curves.

Table S9. Thirty-eight very rare (PopMax FAF<0.2%) LoF or predicted deleterious missense heterozygous variants in 32 individuals with 22q11.2DS prioritized for assessment of clinical relevance with respect to extreme levels of TG, LDLC or HDLC.

| **ID** | **Sex** | **Age (recent)^a^** | **Age (max)^b^** | **On statins** | **Chr** | **Gene** | **Exon** | **DNA change** | **Amino acid change** | **Variant effect** | **PopMax FAF^c^** | **Associated lipid trait** | **Lipid level (recent) (mmol/L)^a^** | **Lipid level (max) (mmol/L)^b^** | **ACMG Classification** |
| --- | --- | --- | --- | --- | --- | --- | --- | --- | --- | --- | --- | --- | --- | --- | --- |
| 1 | M | 36.7 | 36.7 | N | 7 | *MLXIPL* | 14 | c.G2161A | p.E721K | Missense | 0.001342 | High TG | 4.04 | 4.04 | NA |
| 2 | F | 38.3 | 38.3 | N | 11 | *APOA4* | 3 | c.C476G | p.P159R | Missense | 0.000003 | High TG | 1.30 | 1.30 | VUS |
| 3 | F | 25.2 | 25.2 | N | 11 | *APOA4* | 3 | c.A1054T | p.N352Y | Missense | 0.000420 | High TG | 0.43 | 0.43 | VUS |
| 4 | M | 41.4 | 26.5 | N | 12 | *GPD1* | 4 | c.G292A | p.G98R | Missense | 0.001817 | High TG | 1.36 | 2.91 | NA |
| 5 | F | 21.4 | 20.4 | N | 12 | *GPD1* | 4 | c.T431C | p.M144T | Missense | 0.000927 | High TG | 3.67 | 5.37 | NA |
| 6 | M | 23.1 | 21.9 | Y | 16 | *LMF1* | 9 | c.G493A | p.V165M | Missense | 0.000153 | High TG | 7.31 | 11.26 | VUS |
| 7 | M | 20.3 | 20.3 | N | 16 | *LMF1* | 11 | c.C1567T | p.R523C | Missense | 0.000031 | High TG | 1.52 | 1.52 | VUS |
| 8 | F | 18 | 18 | Y | 16 | *LMF1* | 6 | c.T848A | p.L283H | Missense | 0 | High TG | 1.37 | 1.37 | VUS |
| 9 | F | 47.4 | 46.9 | Y | 19 | *CREB3L3* | 6 | c.729dupG | p.L243fs | Frameshift insertion | 0.001025 | High TG | 1.57 | 3.89 | **Likely Pathogenic** |
| 9 | F | 47.4 | 46.9 | Y | 19 | *CREB3L3* | 6 | c.A735T | p.K245N | Missense | 0.001026 | High TG | 1.57 | 3.89 | VUS |
| 11 | M | 41.3 | 41.3 | N | 19 | *CREB3L3* | 8 | c.C934T | p.R312X | Stopgain | 0.001180 | High TG | 2.29 | 2.29 | VUS |
| 12 | F | 42.2 | 42.2 | N | 22 | *PPARA* | 4 | c.A8G | p.D3G | Missense | 0.000022 | High TG | 1.02 | 1.02 | NA |
| 13 | M | 21.1 | 21.1 | N | 22 | *PPARA* | 4 | c.C40T | p.L14F | Missense | 0.000003 | High TG | 4.23 | 4.23 | NA |
| 14 | M | 23.9 | 23.9 | N | 2 | *ABCG5* | 1 | c.G116A | p.G39D | Missense | 0.000018 | High LDLC | 2.54 | 2.54 | VUS |
| 15 | F | 22.1 | 22.1 | N | 2 | *ABCG8* | 7 | c.G977T | p.S326I | Missense | 0.000023 | High LDLC | 2.47 | 2.47 | NA |
| 16 | M | 35.2 | 28.8 | N | 2 | *ABCG8* | 6 | c.C860T | p.T287M | Missense | 0.000017 | High LDLC | 2.35 | 2.60 | NA |
| 17 | M | 53.1 | 51 | N | 2 | *ABCG8* | 11 | c.C1666G | p.H556D | Missense | 0 | High LDLC | 2.97 | 2.99 | NA |
| 18 | M | 20.3 | 20.3 | N | 2 | *ABCG8* | 9 | c.A1222G | p.N408D | Missense | 0.000563 | High LDLC | 1.77 | 1.77 | NA |
| 19 | M | 23.5 | 23.5 | N | 2 | *ABCG8* | 11 | c.G1720A | p.G574R | Missense | 0.000054 | High LDLC | 2.59 | 2.59 | NA |
| 20 | M | 23 | 23 | N | 2 | *APOB* | 26 | c.C7841T | p.T2614I | Missense | 0 | High LDLC | 2.99 | 2.99 | VUS |
| 21 | F | 31.6 | 20.7 | N | 2 | *APOB* | 26 | c.G6017T | p.G2006V | Missense | 0 | High LDLC | 1.90 | 1.93 | VUS |
| 24 | F | 26.9 | 26.9 | N | 10 | *LIPA* | 10 | c.C1007T | p.P336L | Missense | 0.000052 | High LDLC | 3.53 | 3.53 | VUS |
| 13 | M | 21.1 | 20.7 | N | 19 | *LDLR* | 4 | c.G523A | p.D175N | Missense | 0.000019 | High LDLC | 4.53 | 5.13 | **Pathogenic** |
| 26 | F | 55 | 55 | N | 9 | *ABCA1* | 46 | c.C6083T | p.A2028V | Missense | 0.000267 | Low HDLC | 1.30 | 1.30 | VUS |
| 34 | F | 39.7 | 39.4 | N | 9 | *ABCA1* | 30 | c.C4249T | p.R1417C | Missense | 0.000023 | Low HDLC | 1.55 | 1.60 | VUS |
| 28 | F | 59.4 | 59.4 | Y | 9 | *ABCA1* | 21 | c.A3053G | p.D1018G | Missense | 0.000797 | Low HDLC | 1.17 | 1.17 | VUS |
| 21 | F | 31.6 | 20.7 | N | 9 | *ABCA1* | 25 | c.C3542T | p.S1181F | Missense | 0.001800 | Low HDLC | 1.56 | 1.61 | VUS |
| 30 | M | 57.8 | 55.1 | Y | 9 | *ABCA1* | 25 | c.C3542T | p.S1181F | Missense | 0.001800 | Low HDLC | 0.88 | 1.13 | VUS |
| 31 | F | 52.8 | 52.8 | N | 11 | *APOA1* | 4 | c.A209G | p.H70R | Missense | 0 | Low HDLC | 1.20 | 1.20 | VUS |
| 32 | F | 51.8 | 51.8 | Y | 21 | *ABCG1* | 13 | c.G1553A | p.R518H | Missense | 0 | Low HDLC | 1.70 | 1.70 | NA |
| 33 | F | 17.2 | 17.2 | N | 15 | *LIPC* | 6 | c.C968T | p.T323M | Missense | 0.000478 | High HDLC | 0.80 | 0.80 | VUS |
| 34 | F | 39.7 | 39.4 | N | 15 | *LIPC* | 6 | c.G862A | p.D288N | Missense | 0 | High HDLC | 1.55 | 1.60 | VUS |
| 14 | M | 23.9 | 23.9 | N | 15 | *LIPC* | 3 | c.C403T | p.R135C | Missense | 0.001575 | High HDLC | 1.20 | 1.20 | VUS |
| 32 | F | 51.8 | 51.8 | Y | 16 | *CETP* | 9 | c.G818A | p.G273E | Missense | 0.000017 | High HDLC | 1.70 | 1.70 | VUS |
| 37 | M | 26.1 | 22.1 | N | 18 | *LIPG* | 6 | c.A964G | p.N322D | Missense | 0.000010 | High HDLC | 1.07 | 1.94 | VUS |
| 38 | F | 18.4 | 18.4 | N | 18 | *LIPG* | 9 | c.C1426T | p.R476W | Missense | 0.001941 | High HDLC | 1.21 | 1.21 | VUS |
| 39 | F | 30.9 | 24.9 | N | 18 | *LIPG* | 8 | c.C1426T | p.R476W | Missense | 0.001941 | High HDLC | 1.38 | 2.29 | VUS |
| 40 | F | 36.8 | 23.7 | N | 18 | *LIPG* | 9 | c.C1426T | p.R476W | Missense | 0.001941 | High HDLC | 1.42 | 1.67 | VUS |

F, female; M, male; Y, yes; N, no; TG, triglyceride; LDLC, low density lipoprotein cholesterol; HDLC, high density lipoprotein cholesterol; TC, total cholesterol; chr, chromosome; PopMax FAF, population maximum filtering allele frequency; ACMG, American College of Medical Genetics and Genomics; NA, not applicable; VUS, variant of uncertain significance. All variants presented are heterozygous.

^a^Based on the most recent available measurement (i.e., the measurement used in all other analyses in this study).

^b^Based on the lifetime maximum measurement of a lipid level (used only in this analysis).

^c^gnomAD population maximum filtering allele frequency (<http://cardiodb.org/allelefrequencyapp/>).

**Table S10.** Variant assessments based on ACMG/AMP guidelines for the two variants classified as pathogenic/likely pathogenic.

| **VARIANT 1:**  *LDLR*:NM_000527:exon4:c.G523A:p.D175N; genomic coordinate: chr19:11216105:G:A; dbSNP ID: rs121908033; ClinVar ID: 3726  **Phenotype info of affected individual:**  21.1-year-old male, European ancestry (half-dutch)  LDL cholesterol: 4.53 mmol/L (most recent, age 21.1 years); 5.13 mmol/L (max, age 20.7 years)  BMI: 19.9 (age 21.2 years)  No record of being on statin medication at the time of study. | | |
| --- | --- | --- |
| **Criteria satisfied (Chora et al. 2022)**[5] | **Variant info** | |
| **PS4** - Variant is found in ≥10 unrelated FH cases (FH diagnosis met by validated clinical criteria). | Vergotine et al. 2001 (PMID: 11845603) identified this variant in 31 FH index cases in a South African sample (all cases must have total cholesterol in ≥90 percentile by age and sex, and clinical features of FH (eg Xanthoma, or family history of early CHD). This variant was then identified in 55 family members of these index cases, of which 97% had total cholesterol > 80^th^ percentile and 83% > 95^th^ percentile. | |
| **PM1** - Missense variant located in exon 4, or a missense change in 1 of 60 highly conserved cysteine residues | LDLR:NM_000527:exon4:c.G523A:p.D175N | |
| **PM2** - Variant has a PopMax MAF ≤ 0.0002 (0.02%) in gnomAD. Consider exceptions for known founder variants | PopMax MAF=0.0001088 in East Asian, MAF=0 in non-Finnish Europeans | |
| **PS3_Moderate** - Variant meets level 2 pathogenic functional study criteria ((1) Study of only part of the LDLR cycle following level 1 methodology or whole or part of the LDLR cycle in true homozygous patient cells. Assay results of <70% of wild-type activity in either LDLR expression/ biosynthesis, LDL binding, or LDL internalization) | Van Roggen et al. 1995 PMID: 7773731 re-created the mutation through site-directed mutagenesis. Mutant receptors had only 20% of LDL binding capacity vs WT and decreased cell surface expression and turnover rate. Receptor internalization was not studied (not eligible for PS3 level 1). | |
| **PP3** - REVEL score ≥ 0.75 (missense variants) or predicted impact to splicing using  MaxEntScan | REVEL score=0.801 | |
| **PS3_Supporting** - Variant meets level 3 pathogenic functional study criteria ((1) Study of LDLR cycle (whole or part) in heterozygous patient cells. Assay results of <85% of wild-type activity in either LDLR expression/biosynthesis, LDL binding, or LDL internalization) | Van Roggen et al. 1995 PMID: 7773731 Similar results as listed in PS3_Moderate were found when using heterozygous patient cells | |
| **Conclusion**: 1 strong + 3 moderate + 2 supporting 🡪 pathogenic with respect to familial hypercholesterolemia. | | |
|  | | |
| **VARIANT 2:**  *CREB3L3:*NM_001271995:exon6:c.729dupG:p.L243fs; genomic coordinate: chr19:4168364:T:TG; dbSNP ID: rs780374391; ClinVar ID: 967101  **Phenotype info of affected individual:**  47.4 year old female, European ancestry  Triglyceride: 1.57 mmol/L (most recent, age 47.4 years), 3.89 mmol/L (max, 46.9 years)  BMI: 37 (age 47 years)  Record of being on statin medication | | |
| **Criteria satisfied** (Richards et al. 2015)[6] | | **Variant info** |
| **PVS1** null variant (nonsense, frameshift, canonical ±1 or 2 splice sites, initiation codon, single or multiexon deletion) in a gene where LOF is a known mechanism of disease | | This is a frameshift loss of function variant.  Multiple lines of evidence support *CREB3L3* being associated with elevated triglyceride (TG) levels. Individuals with severe hypertriglyceridemia were found to be 20X more likely to carry a heterozygous LOF variant in *CREB3L3* (Dron et al 2020; 32580631). *CREB3L3* was associated with higher TG levels, reaching exome-wide significance in a large lipids exome-sequencing study (Hindy et al. 2022). CREB-H-deficient mice showed hypertriglyceridemia due to inefficient triglyceride clearance (Lee et al. 2011; 21666694). This variant is also found at a low frequency of 0.0004899 among non-Finnish EUA in gnomAD (PM2) |
| **PM2** Absent from controls (or at extremely low frequency if recessive) (table 6) in Exome Sequencing Project, 1000 Genomes Project, or Exome Aggregation Consortium | | gnomAD popmax filtering AF: 0.001025  gnomAD non-Finnish European: 0.0004899  These frequencies are below the 0.2% cut-off defined for this study. |
| **Conclusion:** 1 very strong + 1 moderate 🡪 Likely pathogenic with respect to mild-moderate HTG | | |

**Table S11.** Variants (frequency<1.0%) in adults with 22q11.2DS included in the rare variant burden analyses.

| **Coordinate** | **Variant type** | **Splice Type^a^** | **snv_type** | **refseq_id** | **gene_symbol** | **entrez_id** | **gnomAD PopMax FAF** | **dbsnp** | **associated_lipid_trait^b^** |
| --- | --- | --- | --- | --- | --- | --- | --- | --- | --- |
| 15:58840688:C:T | exonic | non-splice | missense | NM_000236:exon6:c.C968T:p.T323M | LIPC | 3990 | 0.00047798 | rs573111255 | HDLC_high |
| 15:58840582:G:A | exonic | non-splice | missense | NM_000236:exon6:c.G862A:p.D288N | LIPC | 3990 | 0 | rs761575901 | HDLC_high |
| 15:58834113:C:T | exonic | non-splice | missense | NM_000236:exon3:c.C403T:p.R135C | LIPC | 3990 | 0.00157487 | rs201014789 | HDLC_high |
| 15:58855772:T:C | exonic | non-splice | missense | NM_000236:exon8:c.T1238C:p.L413P | LIPC | 3990 | 0.00416866 | rs569503516 | HDLC_high |
| 18:47107955:A:G | exonic | non-splice | missense | NM_006033:exon6:c.A964G:p.N322D | LIPG | 9388 | 9.58E-06 | rs755737325 | HDLC_high |
| 18:47113165:C:T | exonic | non-splice | missense | NM_006033:exon9:c.C1426T:p.R476W | LIPG | 9388 | 0.00194058 | rs117623631 | HDLC_high |
| 18:47113165:C:T | exonic | non-splice | missense | NM_001308006:exon8:c.C1204T:p.R402W | LIPG | 9388 | 0.00194058 | rs117623631 | HDLC_high |
| 18:47113165:C:T | exonic | non-splice | missense | NM_006033:exon9:c.C1426T:p.R476W | LIPG | 9388 | 0.00194058 | rs117623631 | HDLC_high |
| 12:125294817:C:A | exonic;splicing | non-canonical | missense | NM_001367984:exon6:c.G745T:p.D249Y | SCARB1 | 949 | 7.42E-05 | rs201357313 | HDLC_high |
| 12:125294817:C:A | exonic;splicing | non-canonical | missense | NM_001367987:exon6:c.G745T:p.D249Y | SCARB1 | 949 | 7.42E-05 | rs201357313 | HDLC_high |
| 9:107550322:G:A | exonic | non-splice | missense | NM_005502:exon46:c.C6083T:p.A2028V | ABCA1 | 19 | 0.00026706 | rs200788099 | HDLC_low |
| 9:107599855:A:G | splicing | non-canonical | LOF | NM_005502:exon10:c.1055-7T>C | ABCA1 | 19 | 0.0060995 | rs199586194 | HDLC_low |
| 9:107571772:G:A | exonic | non-splice | missense | NM_005502:exon30:c.C4249T:p.R1417C | ABCA1 | 19 | 2.30E-05 | rs749089795 | HDLC_low |
| 9:107582258:T:C | exonic | non-splice | missense | NM_005502:exon21:c.A3053G:p.D1018G | ABCA1 | 19 | 0.00079709 | rs140365800 | HDLC_low |
| 9:107589238:C:G | exonic | non-splice | missense | NM_005502:exon16:c.G2328C:p.K776N | ABCA1 | 19 | 0.00997774 | rs138880920 | HDLC_low |
| 9:107578620:G:A | exonic | non-splice | missense | NM_005502:exon25:c.C3542T:p.S1181F | ABCA1 | 19 | 0.00179963 | rs76881554 | HDLC_low |
| 9:107578620:G:A | exonic | non-splice | missense | NM_005502:exon25:c.C3542T:p.S1181F | ABCA1 | 19 | 0.00179963 | rs76881554 | HDLC_low |
| 9:107599385:C:T | splicing | non-canonical | LOF | NM_005502:exon11:c.1195-8G>A | ABCA1 | 19 | 0.0001904 | rs201011354 | HDLC_low |
| 11:116706792:T:C | exonic | non-splice | missense | NM_001318021:exon4:c.A209G:p.H70R | APOA1 | 335 | 0 | rs1023694493 | HDLC_low |
| 2:21231899:G:A | exonic | non-splice | missense | NM_000384:exon26:c.C7841T:p.T2614I | APOB | 338 | 0 | rs761038950 | LDLC |
| 2:21231278:G:A | exonic | non-splice | missense | NM_000384:exon26:c.C8462T:p.P2821L | APOB | 338 | 0.00767598 | rs72653095 | LDLC |
| 2:21234674:C:T | exonic | non-splice | missense | NM_000384:exon26:c.G5066A:p.R1689H | APOB | 338 | 0.00298509 | rs151009667 | LDLC |
| 2:21231278:G:A | exonic | non-splice | missense | NM_000384:exon26:c.C8462T:p.P2821L | APOB | 338 | 0.00767598 | rs72653095 | LDLC |
| 2:21233723:C:A | exonic | non-splice | missense | NM_000384:exon26:c.G6017T:p.G2006V | APOB | 338 | 0 | NA | LDLC |
| 19:11222182:T:C | splicing | non-canonical | LOF | NM_001195799:exon7:c.938-8T>C | LDLR | 3949 | 0.00756171 | rs72658861 | LDLC |
| 19:11227525:G:A | splicing | non-canonical | LOF | NM_001195799:exon11:c.1583-10G>A | LDLR | 3949 | 0.0029114 | rs17248882 | LDLC |
| 19:11240355:C:T | splicing | non-canonical | LOF | NM_001195799:exon16:c.2424+9C>T | LDLR | 3949 | 2.60E-05 | rs746674813 | LDLC |
| 19:11216105:G:A | exonic | non-splice | missense | NM_000527:exon4:c.G523A:p.D175N | LDLR | 3949 | 1.90E-05 | rs121908033 | LDLC |
| 19:11222182:T:C | splicing | non-canonical | LOF | NM_001195799:exon7:c.938-8T>C | LDLR | 3949 | 0.00756171 | rs72658861 | LDLC |
| 19:11224206:C:G | splicing | non-canonical | LOF | NM_001195799:exon9:c.1236-5C>G | LDLR | 3949 | 2.93E-06 | rs531005522 | LDLC |
| 19:11222182:T:C | splicing | non-canonical | LOF | NM_001195799:exon7:c.938-8T>C | LDLR | 3949 | 0.00756171 | rs72658861 | LDLC |
| 19:11227525:G:A | splicing | non-canonical | LOF | NM_001195799:exon11:c.1583-10G>A | LDLR | 3949 | 0.0029114 | rs17248882 | LDLC |
| 19:11222182:T:C | splicing | non-canonical | LOF | NM_001195799:exon7:c.938-8T>C | LDLR | 3949 | 0.00756171 | rs72658861 | LDLC |
| 8:144297123:C:T | splicing | non-canonical | LOF | NM_001301772:exon4:c.296-11C>T | GPIHBP1 | 338328 | 0.00160361 | rs201591245 | Triglycerides |
| 8:144297125:C:T | splicing | non-canonical | LOF | NM_001301772:exon4:c.296-9C>T | GPIHBP1 | 338328 | 0.00160335 | rs201256271 | Triglycerides |
| 8:144297123:C:T | splicing | non-canonical | LOF | NM_001301772:exon4:c.296-11C>T | GPIHBP1 | 338328 | 0.00160361 | rs201591245 | Triglycerides |
| 8:144297125:C:T | splicing | non-canonical | LOF | NM_001301772:exon4:c.296-9C>T | GPIHBP1 | 338328 | 0.00160335 | rs201256271 | Triglycerides |
| 16:920817:C:T | exonic | non-splice | missense | NM_001352017:exon9:c.G493A:p.V165M | LMF1 | 64788 | 0.00015266 | rs370036895 | Triglycerides |
| 16:983990:C:T | splicing | canonical | LOF | NM_001352018:exon4:c.115+1G>A | LMF1 | 64788 | 0.00315856 | rs72759474 | Triglycerides |
| 16:904669:G:A | exonic | non-splice | missense | NM_022773:exon11:c.C1567T:p.R523C | LMF1 | 64788 | 3.09E-05 | rs758116895 | Triglycerides |
| 16:929619:A:T | exonic | non-splice | missense | NM_001352020:exon6:c.T848A:p.L283H | LMF1 | 64788 | 0 | NA | Triglycerides |
| 8:19818607:A:G | splicing | non-canonical | LOF | NM_000237:exon8:c.1322+13A>G | LPL | 4023 | 0 | NA | Triglycerides |

^a^Non-canonical splice variants were called by Splice AI[7].

^b^Variants were included in rare variant burden analyses for each associated lipid trait.

**Table S12.** Linear regression analyses testing lipid trait-specific rare carrier status as a predictor for its corresponding lipid level in a univariable model and in a multivariable model accounting for phenotypic, batch, ancestry variables, in adults with 22q11.2DS.

|  | **TG (n=149)^a^** | | | **HDLC (low) (n=150)** | | | **HDLC (high) ( (n=150)** | | | **LDLC (n=148)^b^** | | |
| --- | --- | --- | --- | --- | --- | --- | --- | --- | --- | --- | --- | --- |
|  | **beta** | **std error** | **p** | **beta** | **std error** | **p** | **beta** | **std error** | **p** | **beta** | **std error** | **p** |
| **Univariable model** | | | | | | | | | | | |  |
| Rare variant burden^c^ | 0.0808 | 0.0822 | 0.3280 | 0.0954 | 0.0818 | 0.2460 | 0.0884 | 0.0819 | 0.2820 | -0.0789 | 0.0825 | 0.3400 |
| **Multivariable model** | | | | | | | | | | | |  |
| Rare variant burden^c^ | 0.0493 | 0.0829 | 0.5537 | -0.0232 | 0.0742 | 0.7550 | -0.0129 | 0.0745 | 0.8620 | -0.0748 | 0.0863 | 0.3880 |
| Sex | -0.2730 | 0.0810 | **0.0010** | 0.4797 | 0.0730 | **9.39E-10** | 0.4787 | 0.0731 | **1.08E-09** | 0.0761 | 0.0879 | 0.3880 |
| Age | 0.0010 | 0.0876 | 0.9905 | 0.0675 | 0.0803 | 0.4020 | 0.0608 | 0.0779 | 0.4360 | 0.0694 | 0.0934 | 0.4590 |
| BMI | 0.2375 | 0.0833 | **0.0050** | -0.3018 | 0.0745 | **8.55E-05** | -0.3003 | 0.0743 | **8.85E-05** | 0.0654 | 0.0901 | 0.4690 |
| T2D | -0.0421 | 0.0826 | 0.6114 | 0.0952 | 0.0739 | 0.2000 | 0.0950 | 0.0740 | 0.2020 | -0.0286 | 0.0886 | 0.7470 |
| Psychotic^d^ | 0.0135 | 0.0830 | 0.8709 | -0.0003 | 0.0741 | 0.9960 | -0.0012 | 0.0745 | 0.9880 | 0.0486 | 0.0904 | 0.5920 |
| Cohort | -0.0636 | 0.1640 | 0.6987 | 0.1685 | 0.1468 | 0.2530 | 0.1685 | 0.1473 | 0.2550 | 0.0742 | 0.1755 | 0.6730 |
| Sequencing Platform | 0.1940 | 0.1301 | 0.1382 | -0.1472 | 0.1164 | 0.2080 | -0.1482 | 0.1171 | 0.2080 | -0.0942 | 0.1393 | 0.5000 |
| PC1 | -0.0383 | 0.0831 | 0.6462 | -0.0041 | 0.0736 | 0.9550 | -0.0064 | 0.0748 | 0.9320 | -0.0266 | 0.0889 | 0.7660 |
| PC2 | -0.0397 | 0.1046 | 0.7052 | 0.0903 | 0.0914 | 0.3250 | 0.0933 | 0.0936 | 0.3210 | 0.0774 | 0.1103 | 0.4840 |
| PC3 | 0.1566 | 0.1480 | 0.2919 | 0.0003 | 0.1327 | 0.9980 | 0.0007 | 0.1329 | 0.9960 | -0.0809 | 0.1588 | 0.6110 |
| PC4 | 0.0178 | 0.0898 | 0.8436 | -0.0128 | 0.0806 | 0.8750 | -0.0147 | 0.0803 | 0.8550 | -0.0742 | 0.0966 | 0.4440 |
|  |  | **R^2^** | **p** |  | **R^2^** | **p** |  | **R^2^** | **p** |  | **R^2^** | **p** |
| Model |  | 0.1652 | **0.0129** |  | 0.3269 | **1.13E-07** |  | 0.3266 | **1.16E-07** |  | 0.0358 | 0.9543 |

TG, triglyceride; LDLC, low density lipoprotein cholesterol; HDLC, high density lipoprotein cholesterol; TC, total cholesterol; BMI, body mass index; T2D, type 2 diabetes.

Beta coefficients are standardized with positive values indicating a positive association between higher lipid levels and female sex, older age, higher BMI, having type 2 diabetes, having a psychotic illness, belonging to the TCAG cohort, sequenced on HiSeq X, and higher PCA, except for rare carrier status and low HDLC where a positive beta value would indicate association with lower HDLC levels.

^a^Excluded one individual on fibrate treatment. Triglyceride levels were natural log transformed to approximate a normal distribution.

^b^For individuals on statin medications, LDLC and TC levels were divided by 0.7 and 0.8, respectively.

^c^The presence of one or more rare (PopMax FAF<1.0%) variants in a canonical gene associated with the lipid trait of interest.

^d^Defined as schizophrenia or schizoaffective disorder.

Bold font indicates statistical significance


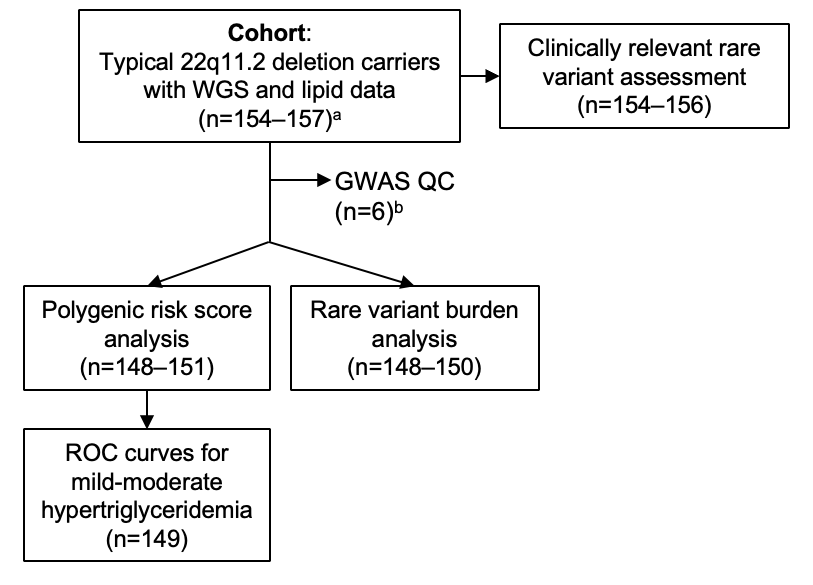


**Figure S1. Overview of study design.**

^a^Consists of individuals with recorded levels of triglycerides (n=156), low density lipoprotein cholesterol (n=154), high density lipoprotein cholesterol (n=156), and total cholesterol (n=157). All individuals are contained within the 157 individuals with total cholesterol levels.

^b^Consists of excluded for being related to another individual in the cohort (n=3) and outlying heterozygosity (n=3). One additional individual on fibrate medication was excluded for subsequent TG analyses only.

**Figure S2.** First two principal components of individuals with 22q11.2DS from the TCAG (n=88) and IBBC (n=63) cohorts, and individuals from 1000 Genomes with assigned ancestry. A cut-off of -0.02 on the PC2 axis was used to assign individuals in this study (TCAG or IBBC) as European (<-0.02) or non-European (>0.02).


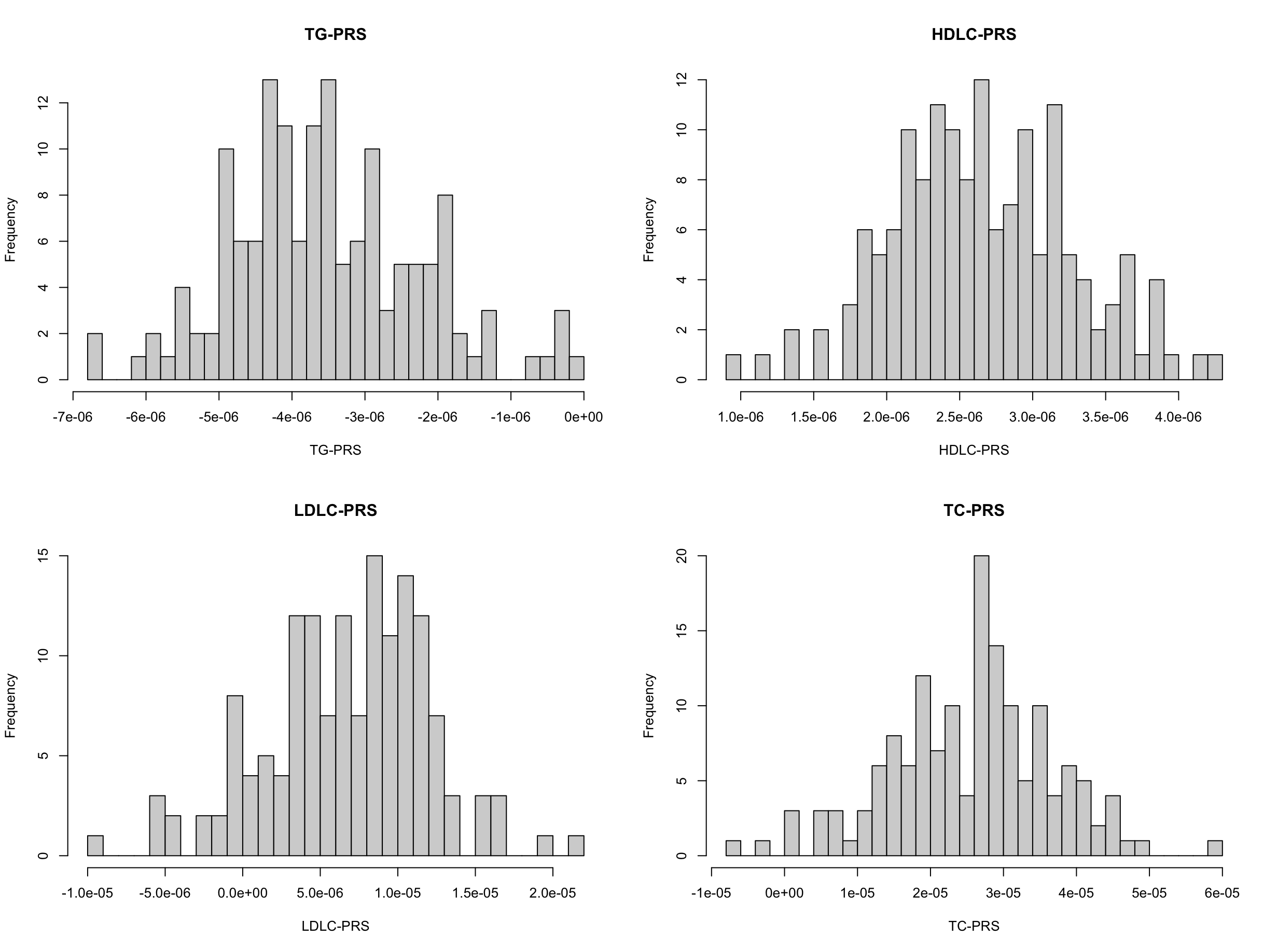


**Figure S3.** Distribution of polygenic risk scores (PRS) for triglycerides (TG) (n=149), high density lipoprotein cholesterol (HDLC) (n=150), low density lipoprotein cholesterol (LDLC) (n=148), and total cholesterol (TC) (n=151).


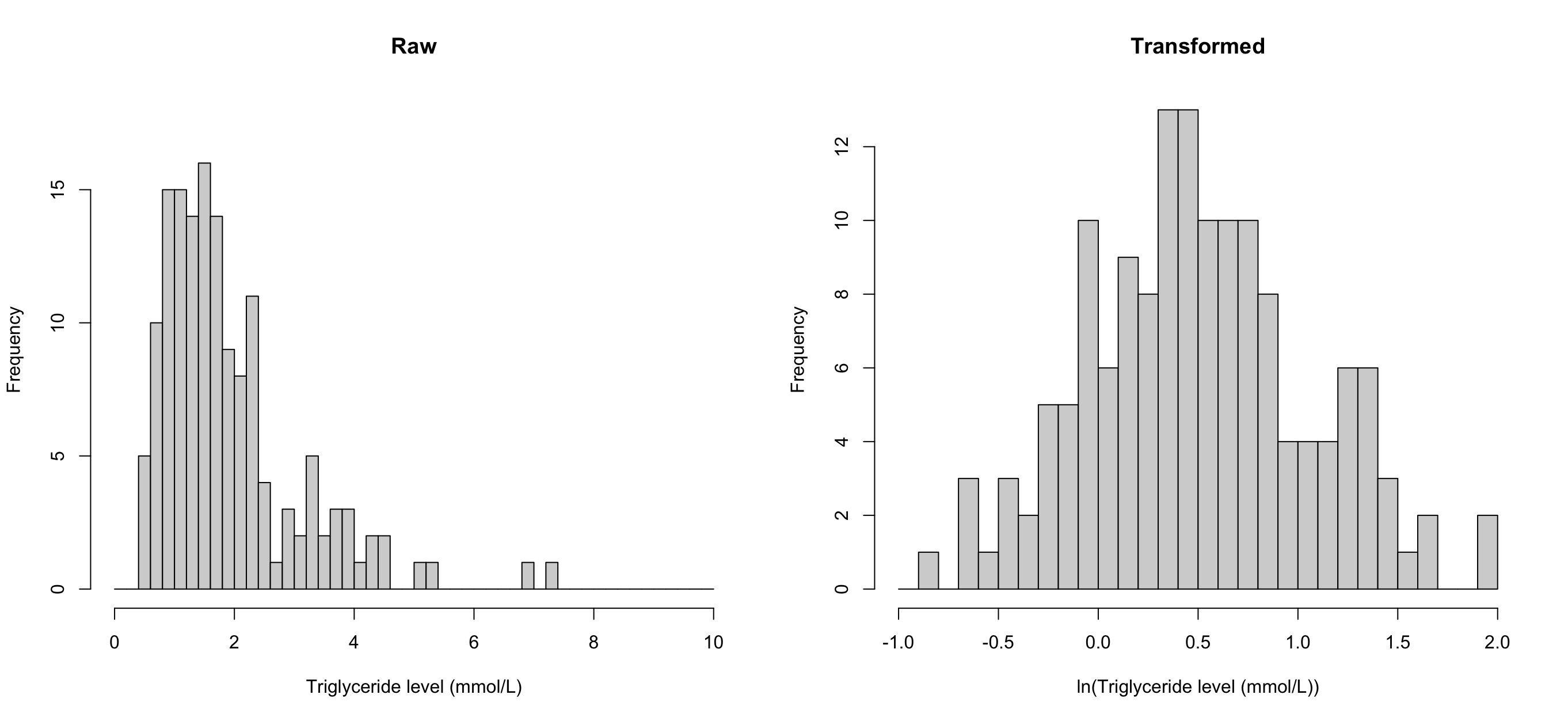


**Figure S4**. Distribution of triglyceride levels in individuals with a 22q11.2 deletion (n=149), before and after log transformation.

**Figure S5.** Number of rare variants called per individual between the three site-platform groups, by variant type, for n=151 individuals included in the rare variant burden analyses. The mean ± SD number of variants across all three groups (n=151) is displayed next to each plot title. Variant count means between the three site-platform groups were compared using the Kruskal–Wallis test and pairwise comparisons were made using Wilcoxon signed-rank without adjustment for multiple comparisons. Samples were sequenced to average ± standard deviation (SD) read depths of 29.36 ± 8.42 (IBBC-HiSeq 2500), 37.22 ± 10.90 (IBBC-HiSeq X) and 94.58 ± 23.33 (TCAG-HiSeq X). The read depths reported for the TCAG-HiSeq X samples are the combined read depths of the proband and both parents.

**Figure S6.** Lipid levels of individuals from each site-platform group for n=151 individuals included in the polygenic risk score and rare variant burden analyses. Lipid levels between the three site-platform groups per trait were compared using the Kruskal-Wallis test and pairwise comparisons were made using Wilcoxon signed-rank without adjustment for multiple comparisons. TG, triglyceride; LDLC, low density lipoprotein cholesterol; HDLC, high density lipoprotein cholesterol; TC, total cholesterol.


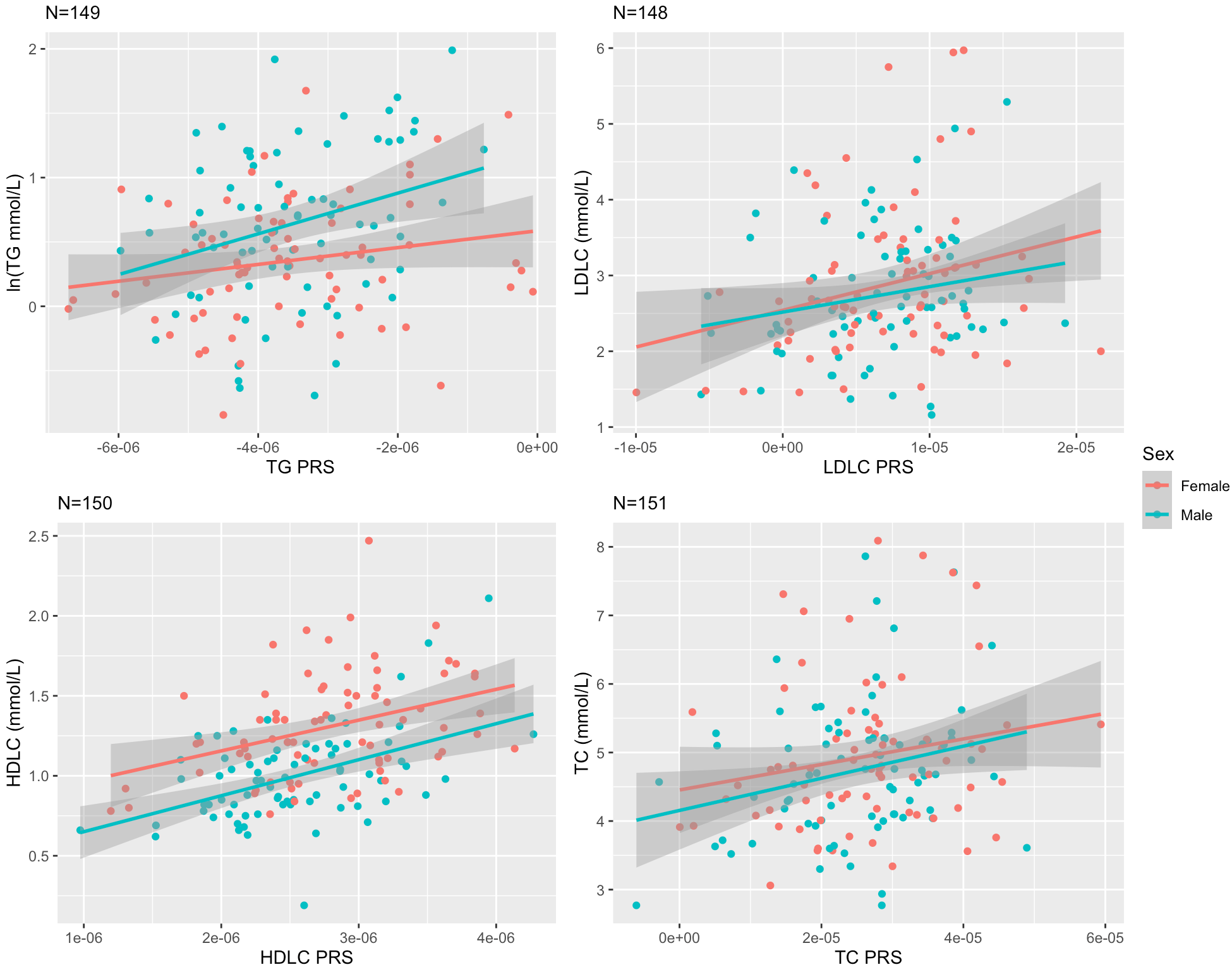


**Figure S7. Linear association between the polygenic risk score (common variants) for each lipid trait and its corresponding lipid level.** Fitted lines were generated using linear regression performed within sex. TG, triglyceride; LDLC, low density lipoprotein cholesterol; HDLC, high density lipoprotein cholesterol; TC, total cholesterol; PRS, polygenic risk score.

**Figure S8.** Receiver operating characteristic (ROC) curves of logistic regression models predicting mild-moderate hypertriglyceridemia in adults with 22q11.2DS. Each plot contains two ROC curves. One contains only one or two clinical variable(s) as predictors: BMI (A), sex (B) or both (C). The other curve includes the TG-PRS with the respective clinical variable(s). The area under the curve (AUC) and 95% confidence intervals (95% CI) for each curve are shown in the figure key on the bottom right of each plot.

**Figure S9.** The proportion of adult individuals with 22q11.2DS and (A) mild-moderate hypertriglyceridemia (HTG) and (B) the triglyceride (TG) level (mmol/L) for individuals in each decile of triglyceride polygenic risk score (TG-PRS) (n=14–15 per decile; n=149 total). The dashed line in B indicates the lower-bound cut-off for mild-moderate HTG (1.7 mmol/L).

**REFERENCES**

1. Blagojevic C, Heung T, Malecki S, Ying S, Cancelliere S, Hegele RA, et al. Hypertriglyceridemia in young adults with a 22q11.2 microdeletion. Eur J Endocrinol. Bioscientifica Ltd; 2022;187:91–9.

2. Privé F, Aschard H, Carmi S, Folkersen L, Hoggart C, O’Reilly PF, et al. Portability of 245 polygenic scores when derived from the UK Biobank and applied to 9 ancestry groups from the same cohort. Am J Hum Genet. 2022;109:12–23.

3. Lambert SA, Gil L, Jupp S, Ritchie SC, Xu Y, Buniello A, et al. The Polygenic Score Catalog as an open database for reproducibility and systematic evaluation. Nat Genet. Nature Publishing Group; 2021;53:420–5.

4. Dron JS, Wang J, McIntyre AD, Iacocca MA, Robinson JF, Ban MR, et al. Six years’ experience with LipidSeq: clinical and research learnings from a hybrid, targeted sequencing panel for dyslipidemias. BMC Med Genomics. 2020;13:23.

5. Chora JR, Iacocca MA, Tichý L, Wand H, Kurtz CL, Zimmermann H, et al. The Clinical Genome Resource (ClinGen) Familial Hypercholesterolemia Variant Curation Expert Panel consensus guidelines for LDLR variant classification. Genet Med. 2022;24:293–306.

6. Richards S, Aziz N, Bale S, Bick D, Das S, Gastier-Foster J, et al. Standards and guidelines for the interpretation of sequence variants: a joint consensus recommendation of the American College of Medical Genetics and Genomics and the Association for Molecular Pathology. Genet Med. 2015;17:405–23.

7. Jaganathan K, Panagiotopoulou SK, McRae JF, Darbandi SF, Knowles D, Li YI, et al. Predicting splicing from primary sequence with deep learning. Cell. Elsevier; 2019;176:535-548.e24.
