# Supplementary material for: Polygenic risk for triglyceride levels in the presence of a high impact rare variant
Source: BMC Med Genomics. 2023 Nov 8;16:281. doi: 10.1186/s12920-023-01717-2 (PMC10634078; doi:10.1186/s12920-023-01717-2)
Supplement: Supplementary file 2 — Additional file 2. Supplementary Methods. [file 12920_2023_1717_MOESM2_ESM.docx]

**Supplementary Methods**

**Genome sequencing and variant annotation**

Genome sequencing data used for this study was performed at two sites. Ninety individuals were sequenced using the Illumina HiSeq X platform at The Centre for Applied Genomics (TCAG) in Toronto, Canada. Sequencing data for the remaining 67 individuals was derived from either the Illumina HiSeq X (n=34) or Illumina HiSeq 2500 (n= 33) platforms at HudsonAlpha Genome Sequencing Center in Huntsville, United States. These data were generated as part of an International Brain and Behavior Consortium (IBBC) study [1]. For sequencing data independently obtained at both sites (IBBC and TCAG) for 39 individuals, we opted to use data from TCAG due to the ability to refine variant calls using parental data. A comparison of the sequencing metrics, number of rare variants called, and differences in lipid levels between each site-platform group can be found in Additional file 1: Figures S5 and S6.

Detailed sequencing methods for the IBBC samples are described in Cleynen et al. [1] and the methods used for the TCAG samples are identical to those described in Mojarad et al. [2]. Briefly, for both samples, sequenced reads were aligned to the reference genome (GRCh37/hg19) using the Burrows-Wheeler Aligner version 0.7.12 as a sorted binary alignment map (BAM) format. Genome Analysis ToolKit (GATK) (https://gatk.broadinstitute.org/hc/en-us) was used for indel realignment, quality score recalibration and SNV and indel calling, with version 3.7.0 and 4.0 used to call TCAG and IBBC samples, respectively. For the calling of structural variants, CNVs greater than 10kb were called using estimation by read depth with single-nucleotide variants (ERDS) and CNVnator [3,4]. Only CNVs called by both algorithms were included for analyses. Smaller CNVs (i.e. size < 10kb) were detected using Manta3.4 and LUMPY algorithms [5,6]. Variant annotation was re-done, for the purpose of this study, for both cohorts using the same pipeline at TCAG. Annotation for SNVs/indels was performed using ANNOVAR in-house script v.27.6 (hg19). Annotation for CNVs was performed using TCAG annotation pipeline v.1.5.4. Variants located on sex chromosomes or in the 22q11.2 deletion region (chr22:18,912,231–21,465,672 [GRCh37]) were excluded from all analyses.

**Common variant quality control (QC)**

Standard GWAS QC filters were applied, using PLINK v1.9, to the subset of SNPs extracted from sequencing files that were contained in previously constructed polygenic risk scores [7] (see polygenic risk score section of methods). SNPs with a minor allele frequency (MAF) <1%, missingness rate >1%, and Hardy-Weinberg equilibrium exact test p value <1x10^-6^ were excluded. Individuals with a genotype missingness rate >1%, heterozygosity rate that deviated more than three standard deviations from the mean, and pi-hat>0.2 (relatedness test) were excluded. Using these filters, three individuals were excluded from subsequent analyses for outlying heterozygosity and another three were excluded for being related to another member of the cohort (consistent with pedigree information) (Additional file 1: Figure S1). All analyses, except for the assessment of clinically relevant rare variants, were performed within these remaining 148–151 individuals (Additional file 1: Figure S1).

**Principal component analyses (PCA)**

We first restricted the set of SNPs extracted for PRS analyses to SNPs of previously QC’d 1000 Genomes data with assigned ancestry (http://www.tcag.ca/tools/1000genomes.html). The genotype data of individuals from this study were then merged with the QC’d 1000 Genomes data, and SNPs were pruned using the PLINK command --indep-pairwise (200 variant window size, step size of 100 variants, and pruning variants with r^2^>0.1), which left ~56,000 SNPs remaining in each cohort (IBBC and TCAG). Principal components where then generated using the --pca command. The individuals from this study were plotted on top of the previously assigned ancestry of the 1000 Genomes data along the first two PCs to infer ancestry (Additional file 1: Figure S2). Individuals with PC2<-0.02 were assigned to being of European ancestry.

**Clinically relevant rare variants**

To prioritize variants for assessment of clinical relevance with respect to their relationship to causing extreme lipid levels (i.e., high TG, LDLC, HDLC, and low HDLC), we restricted to variants affecting protein coding or splicing regions that are (1) very rare (gnomAD PopMax filtering allele frequency<0.2%), (2) loss of function (LoF) or predicted damaging missense, and (3) within genes relevant to lipid levels that are part of a targeted next generation sequencing (NGS) panel (n=33 candidate genes) used at a specialized genetics clinic for lipid metabolism disorders in London, Ontario [8] (Additional file 1: Table S5). Recently developed guidelines by the Clinical Genome Resource (ClinGen) for the assessment of *LDLR* variants supports the use of a variant having a PopMax filtering allele frequency>0.2% as evidence for the variant being benign [9]. LoF variants were defined as stop-gain, frameshift insertion or deletion (indel), or splice site altering variants. Damaging missense variants were defined as being predicted deleterious by four or more of eight in silico programs ([CADD (≥15), SIFT (≤0.05), PolyPhen2 HVAR (≥0.90), Provean (≤-2.5), ma (≥1.90) and mt (≥0.5) scores, PhyloPMam (≥2.30) and PhyloPVert (≥4.0)] [10].We selected a total of 33 genes from the LipidSeq NGS panel, consisting of genes that when affected by a rare variant may cause abnormal levels of TG, LDLC, and HDLC (Additional file 1: Table S5). These genes include both “canonical” lipid metabolism genes that define their associated monogenic lipid disorders and for which their involvement in physiological pathways affecting lipid metabolism are well understood (e.g., *LDLR*, *APOB*, *LPL*) [11] as well as genes with more putative evidence for association with one or more lipid traits/disorders that have been implicated through methods such as genome-wide association studies (GWAS), exome sequencing/rare variant association studies and/or functional studies/drug targets (e.g., *PPARA*, *GCKR*) [12–14]. CNVs were restricted to those overlapping an exonic region of a gene belonging to the same panel of genes as the sequence level variants. A frequency filter of <1.0% was applied based on MSSNG parental samples as called by both CNVator and ERDS (https://research.mss.ng/).

Following the above prioritization of all rare variants identified for the 33 candidate genes, we then used the American College of Medical Genetics and Genomics (ACMG) variant interpretation guidelines to assign variants to a five-tier classification system: pathogenic, likely pathogenic, variant of uncertain significance (VUS), likely benign, or benign [15]. For variants in *LDLR*, we used the *LDLR*-specific guidelines developed by ClinGen [9]. Because ACMG guidelines are primarily intended for the assessment of variants in genes that have a known association with the disease of interest, we performed the full assessment only in cases where there was at least putative evidence from the literature that the given pattern of inheritance (i.e., heterozygous or homozygous) of the variant in the particular gene may increase risk for an abnormal lipid level. For example, if a gene is known to impact a lipid level in an autosomal recessive manner and there is not even putative evidence from the literature to suggest that a heterozygous variant in the gene may increase risk, then no further consideration would be given to a heterozygous variant in such a gene, and an assessment was deemed “not applicable”.

**Rare variant status regression analyses**

We sought to assess rare whether being a carrier of a rare variant, including those with potentially smaller effect sizes that are not considered pathogenic/likely pathogenic per ACMG criteria, would be associated with altered lipid levels (Additional file 1: Table S5). We thus proceeded similar to the criteria for assessing clinically pathogenic rare variants, however because variants were not individually adjudicated for these analyses, we restricted to variants only in genes canonically associated with lipid metabolism in the Dron et al. NGS panel [8] (Additional file 1: Table S5) as these would presumably be most likely to exert an impact on lipid levels. We also opted for a more inclusive (PopMax filtering allele frequency<1.0%) frequency filter to increase power and capture variants that may confer a more modest effect on lipid levels that would not be clinically notable. For this analysis specifically, LoF splicing variants were detected using Splice AI which captures more potentially deleterious variants compared to restricting to only canonical ± 1 or 2 splice sites [16]. By design, we intended the presence of a rare variant to affect lipid levels in only one direction per analysis, and therefore included genes that have been associated with increasing TG and LDLC levels, and with increasing or decreasing HDLC levels (Additional file 1: Table S5). We excluded all LoF variants in *APOB* and *PCSK9* due to their association with lower LDLC levels [17–19].

Rare variant status indicated the presence or absence (binary outcome) of one or more rare variants in one or more of the canonical genes associated with the given lipid trait in an individual. An association between rare variant status and lipid levels was assessed using the same univariable and multivariable linear regression models as for PRS analyses, but with the rare variant status variable in place of the PRS variable.

**REFERENCES**

1. Cleynen I, Engchuan W, Hestand MS, Heung T, Holleman AM, Johnston HR, et al. Genetic contributors to risk of schizophrenia in the presence of a 22q11.2 deletion. Mol Psychiatry. 2021;26:4496–510.

2. Mojarad BA, Yin Y, Manshaei R, Backstrom I, Costain G, Heung T, et al. Genome sequencing broadens the range of contributing variants with clinical implications in schizophrenia. Transl Psychiatry. 2021;11:1–12.

3. Abyzov A, Urban AE, Snyder M, Gerstein M. CNVnator: An approach to discover, genotype, and characterize typical and atypical CNVs from family and population genome sequencing. Genome Res. 2011;21:974–84.

4. Zhu M, Need AC, Han Y, Ge D, Maia JM, Zhu Q, et al. Using ERDS to infer copy-number variants in high-coverage genomes. Am J Hum Genet. 2012;91:408–21.

5. Chen X, Schulz-Trieglaff O, Shaw R, Barnes B, Schlesinger F, Källberg M, et al. Manta: rapid detection of structural variants and indels for germline and cancer sequencing applications. Bioinformatics. 2016;32:1220–2.

6. Layer RM, Chiang C, Quinlan AR, Hall IM. LUMPY: a probabilistic framework for structural variant discovery. Genome Biol. 2014;15:R84.

7. Privé F, Aschard H, Carmi S, Folkersen L, Hoggart C, O’Reilly PF, et al. Portability of 245 polygenic scores when derived from the UK Biobank and applied to 9 ancestry groups from the same cohort. Am J Hum Genet. 2022;109:12–23.

8. Dron JS, Wang J, McIntyre AD, Iacocca MA, Robinson JF, Ban MR, et al. Six years’ experience with LipidSeq: clinical and research learnings from a hybrid, targeted sequencing panel for dyslipidemias. BMC Med Genomics. 2020;13:23.

9. Chora JR, Iacocca MA, Tichý L, Wand H, Kurtz CL, Zimmermann H, et al. The Clinical Genome Resource (ClinGen) Familial Hypercholesterolemia Variant Curation Expert Panel consensus guidelines for LDLR variant classification. Genet Med. 2022;24:293–306.

10. Qaiser F, Yin Y, Mervis CB, Morris CA, Klein-Tasman BP, Tam E, et al. Rare and low frequency genomic variants impacting neuronal functions modify the Dup7q11.23 phenotype. Orphanet J Rare Dis. 2021;16:6.

11. Berberich AJ, Hegele RA. The complex molecular genetics of familial hypercholesterolaemia. Nat Rev Cardiol. 2019;16:9–20.

12. Hindy G, Dornbos P, Chaffin MD, Liu DJ, Wang M, Selvaraj MS, et al. Rare coding variants in 35 genes associate with circulating lipid levels—A multi-ancestry analysis of 170,000 exomes. Am J Hum Genet. 2022;109:81–96.

13. Graham SE, Clarke SL, Wu K-HH, Kanoni S, Zajac GJM, Ramdas S, et al. The power of genetic diversity in genome-wide association studies of lipids. Nature. 2021;600:675–9.

14. Schoonjans K, Staels B, Auwerx J. Role of the peroxisome proliferator-activated receptor (PPAR) in mediating the effects of fibrates and fatty acids on gene expression. J Lipid Res. 1996;37:907–25.

15. Richards S, Aziz N, Bale S, Bick D, Das S, Gastier-Foster J, et al. Standards and guidelines for the interpretation of sequence variants: a joint consensus recommendation of the American College of Medical Genetics and Genomics and the Association for Molecular Pathology. Genet Med. 2015;17:405–23.

16. Jaganathan K, Panagiotopoulou SK, McRae JF, Darbandi SF, Knowles D, Li YI, et al. Predicting splicing from primary sequence with deep learning. Cell. 2019;176:535-548.e24.

17. Hegele RA. Plasma lipoproteins: genetic influences and clinical implications. Nat Rev Genet. 2009;10:109–21.

18. Kent ST, Rosenson RS, Avery CL, Chen Y-DI, Correa A, Cummings SR, et al. PCSK9 loss-of-function variants, low-density lipoprotein cholesterol, and risk of coronary heart disease and stroke. Circ Cardiovasc Genet. 2017;10:e001632.

19. Peloso GM, Nomura A, Khera AV, Chaffin M, Won H-H, Ardissino D, et al. Rare protein-truncating variants in APOB, lower low-density lipoprotein cholesterol, and protection against coronary heart disease. Circ Genom Precis Med. 2019;12:e002376.
